# Dandelion extract inhibits triple-negative breast cancer cell proliferation by interfering with glycerophospholipids and unsaturated fatty acids metabolism

**DOI:** 10.3389/fphar.2022.942996

**Published:** 2022-09-06

**Authors:** Shan Wang, Hui-feng Hao, Yan-na Jiao, Jia-lei Fu, Zheng-wang Guo, Yang Guo, Yuan Yuan, Ping-ping Li, Shu-yan Han

**Affiliations:** ^1^ Department of Integration of Chinese and Western Medicine, School of Basic Medical Sciences, Peking University, Beijing, China; ^2^ Key Laboratory of Carcinogenesis and Translational Research (Ministry of Education), Department of Integration of Chinese and Western Medicine, Peking University Cancer Hospital and Institute, Beijing, China

**Keywords:** triple-negative breast cancer, dandelion extract, multi-omics, glycerophospholipid metabolism, choline kinase α

## Abstract

Triple-negative breast cancer (TNBC) is the most aggressive breast cancer subtype with limited treatment options and a poor prognosis. TNBC exists widely reprogrammed lipid metabolism, and its metabolic-associated proteins and oncometabolites are promising as potential therapeutic targets. Dandelion (*Taraxacum mongolicum*) is a classical herbal medicine used to treat breast diseases based on traditional Chinese medicine theory and was reported to have antitumor effects and lipid regulatory capacities. Our previous study showed that dandelion extract was effective against TNBC. However, whether dandelion extract could regulate the lipid metabolisms of TNBC and exert its antitumor effects via interfering with lipids metabolism remained unclear. In this study, an integrated approach combined with network pharmacology and multi-omics techniques (including proteomics, metabolomics, and lipidomics) was performed to investigate the potential regulatory mechanisms of dandelion extract against TNBC. We first determined the antitumor effects of dandelion extract *in vitro* and *in vivo*. Then, network pharmacology analysis speculated the antitumor effects involving various metabolic processes, and the multi-omics results of the cells, tumor tissues, and plasma revealed the changes in the metabolites and metabolic-associated proteins after dandelion extract treatment. The alteration of glycerophospholipids and unsaturated fatty acids were the most remarkable types of metabolites. Therefore, the metabolism of glycerophospholipids and unsaturated fatty acids, and their corresponding proteins CHKA and FADS2, were considered the primary regulatory pathways and biomarkers of dandelion extract against TNBC. Subsequently, experimental validation showed that dandelion extract decreased CHKA expression, leading to the inhibition of the PI3K/AKT pathway and its downstream targets, SREBP and FADS2. Finally, the molecular docking simulation suggested that picrasinoside F and luteolin in dandelion extract had the most highly binding scores with CHKA, indicating they may be the potential CHKA inhibitors to regulate glycerophospholipids metabolisms of TNBC. In conclusion, we confirmed the antitumor effects of dandelion extract against TNBC cells *in vitro* and demonstrated that dandelion extract could interfere with glycerophospholipids and unsaturated fatty acids metabolism via downregulating the CHKA expression and inhibiting PI3K/AKT/SREBP/FADS2 axis.

## Introduction

Breast cancer has surpassed lung cancer as the most commonly diagnosed cancer worldwide with high incidence and mortality in the light of the latest global cancer data in 2020 by the International Agency for Research on Cancer. Approximately 15%–20% of primary breast cancers are triple-negative breast cancers (TNBC), characterized by the absence of estrogen receptor (ER), progesterone receptor, and human epidermal growth factor receptor-2. TNBC has specific clinical features with high invasiveness, high metastatic potential, proneness to relapse, and poor prognosis (Wang et al., 2018; Yin et al., 2020). Due to its molecular and clinical heterogeneity, TNBC lacks highly effective targeted drugs and treatment strategies (Burstein et al., 2015; Bianchini et al., 2016). Consequently, it is necessary and urgent to discover newly specific biomarkers and excavate novel and efficacious alternative medications for TNBC treatment.

Deregulated metabolism frequently exists in primary malignant cancers, and several metabolic pathways underlie cancer initiation and progression (Yoshida, 2015). Breast cancer has metabolic heterogeneity, and its reprogrammed metabolic patterns are different between TNBC and ER^+^ subtypes (Yamashita et al., 2017; Gandhi and Das, 2019). Notably, TNBC also has inherent metabolic heterogeneity (Sun et al., 2020), and a clinical study revealed that 60% of TNBC tumor samples existed significantly upregulated lipids metabolism pathways (Gong et al., 2021). Abnormal lipid metabolism in TNBC could provide biomolecules such as unsaturated fatty acids for phospholipid synthesis and cell membrane formation and generate energy, synergistically promoting and sustaining TNBC progression and survival (Hopperton et al., 2014). Simultaneously, multiple lipid metabolism-associated enzymes and metabolites have significantly altered in TNBC (Hilvo et al., 2011; Tonje et al., 2017). For example, sterol-regulatory element-binding protein (SREBP), a critical regulatory molecule in lipid metabolism, and its downstream fatty acid synthase (FASN), fatty acid desaturase 2 (FADS2) were dramatically increased in the TNBC tissues (Griffiths et al., 2013). Compared to the health, lipids analysis of peripheral blood in TNBC patients suggested prominent metabolic disturbances in choline, sphingolipids, and glycerophospholipids. Among them, triglycerides and lysophosphatidylcholine (LPC) levels were significantly downregulated (Eghlimi et al., 2020). The changed metabolic-associated proteins and metabolites reflected the metabolic vulnerabilities of TNBC. Therefore, they could be considered promising biomarkers for discovering and developing novel therapeutic targets and effective alternative treatments.

Natural products and their derivatives are important sources for discovering new small-molecule compounds for cancer treatment (Li and Weng, 2017)*.* Dandelion, termed *Taraxacum mongolicum*, had been used as a classical herbal medicine based on the traditional Chinese medicine theory in treating mammary diseases, including mammary abscess and hyperplasia of mammary glands (Martinez et al., 2015). Sizeable evidence indicated that dandelion and its components have significant inhibitory effects against various tumor cells (Gauhar et al., 2017; Zhu et al., 2017; Duan et al., 2021)and lipids regulatory impact on normal cells and fat animal models, which is closely related to activating the AMP-activated protein kinase (AMPK) pathway (Liu et al., 2014; Gauhar et al., 2017). For example, dandelion extract significantly reduced adipogenesis and lipid accumulation in 3T3-L1 (González-Castejón et al., 2013), as well as decreased serum levels of cholesterol and triglyceride and liver lipid accumulation in high-fat-fed rabbits (Choi et al., 2010) and rats (Davaatseren et al., 2013). A recent study uncovered the mechanisms of the aqueous extract of dandelion against TNBC via the regulation of a series of biological processes involving cell cycle and metabolism (Qu et al., 2022). Our previous studies revealed that dandelion extract could decrease TNBC cell growth via inducing endoplasmic reticulum stress associated-apoptosis (Li et al., 2017)and inhibit TNBC cell malignant phenotype in tumor-associated macrophages microenvironment (Deng et al., 2021). However, whether dandelion extract could regulate the lipid metabolisms of TNBC and exert its antitumor effects via interfering with lipids metabolism remain unclear and need to elucidate.

**FIGURE 1:**
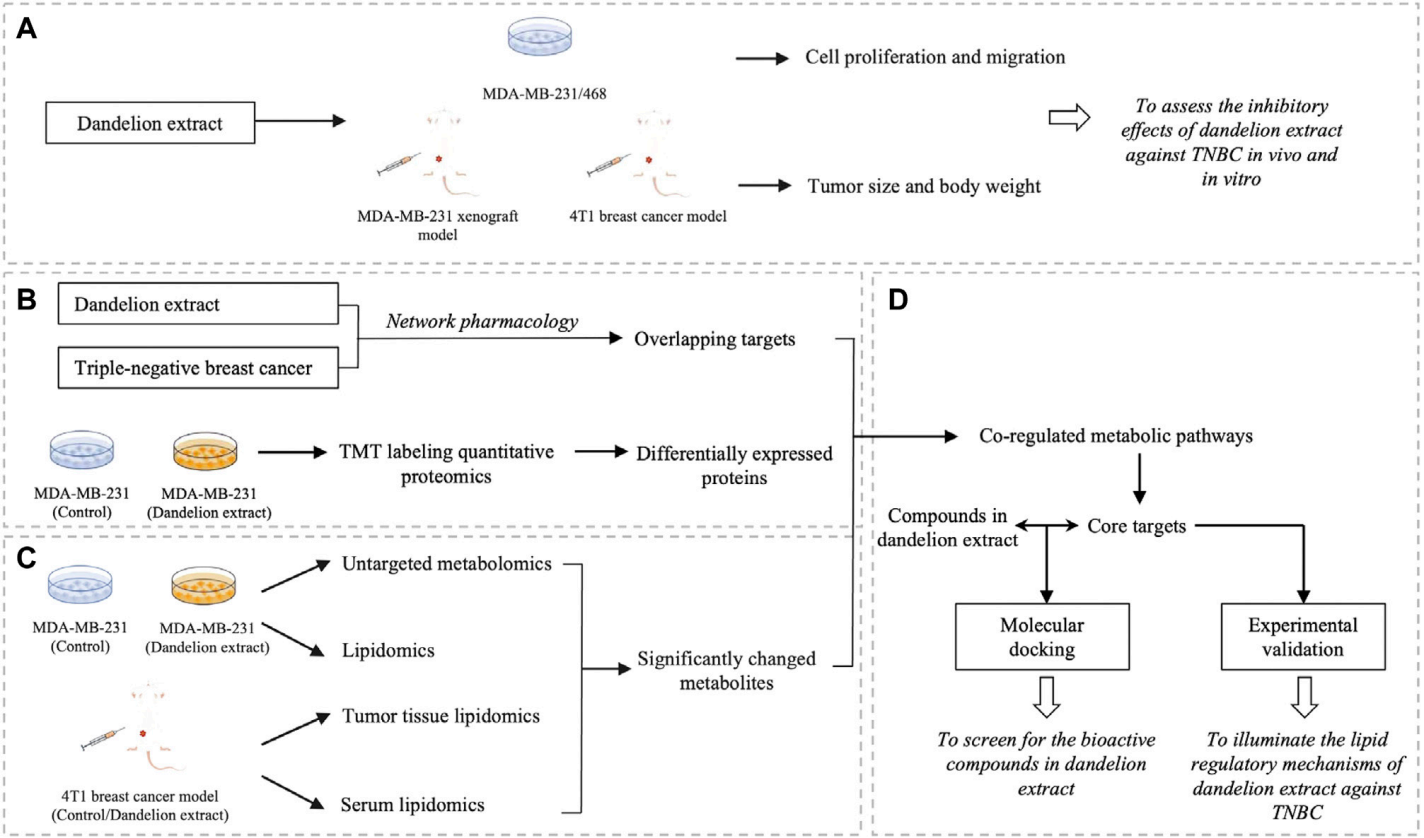
The schematic flowchart of the integrated analysis for dandelion extract against TNBC. **(A)** The inhibitory effects of dandelion extract against TNBC *in vivo* and *in vitro*. **(B)** The Network pharmacology analysis and quantitative proteomic analysis. **(C)** The metabolomics and lipidomics analysis using the TNBC cell, tumor tissue, and plasmas specimens. **(D)** The experimental verification of key targets and the screening for the bioactive compound in dandelion extract via molecular docking.

Network pharmacology is a multidisciplinary approach combining computational biology, network analysis, and experimental verification. It is a promising approach to discovering the underlying mechanisms between the multiple-component drug such as herbal extract and their putative targets (Li and Zhang, 2013; Wang et al., 2021a). Besides, the multi-omics techniques, including genomics, transcriptomics, proteomics, and metabolomics, could directly reflect the changes after drug treatment and have become effective tools to discover potential targets of natural products or TCM (Zhang et al., 2021). Therefore, in the current study, we first demonstrated the antitumor effects of dandelion extract against TNBC *in vitro* and *in vivo*. Subsequently, we conducted an integrated analysis combined with network pharmacology analysis, multi-omics techniques (quantitative proteomics, untargeted metabolomics, and untargeted lipidomics), experimental validation, and molecular docking to investigate the potential regulatory mechanisms of dandelion extract against TNBC *in silico* and *in vitro* and screen for the main bioactive components in dandelion extract. The research flowchart is shown in Figure 1.

## Materials and methods

### Materials and reagents

Fetal bovine serum (FBS), phosphate-buffered saline (PBS, pH 7.2), trypsin, and Dulbecco’s Modified Eagle Medium (DMEM) were purchased from Gibco (Grand Island, NY, United States).3-(4,5-dimethyl thiazol-2-yl-)-2, 5-diphenyl tetrazolium bromide (MTT), and dimethyl sulfoxide (DMSO) were purchased from Sigma-Aldrich (St. Louis, MO, United States). Choline and phosphorylcholine were purchased from TOP SCIENCE (Shanghai, China).

### Cell culture

TNBC cell lines MDA-MB-231 and MDA-MB-468 and human normal mammary epithelial cell MCF-10A were obtained from Peking Union Medical College Cell Bank (Beijing, China). MDA-MB-231 and MDA-MB-468 were cultured in DMEM supplemented with 10% FBS, penicillin (100 units/mL), and streptomycin (100 μg/ml). All cells were maintained in a 5% CO_2_ incubator at 37°C. MCF-10A were cultured in the dedicated medium (Procell, CM-0525, China) with 5%cholera toxin (Macgene, CC104, China).

### Dandelion extract preparation

The dandelion used in this study was identified as *Taraxacum mongolicum* Hand. Mazz, and its extract was produced as the previous report (Li et al., 2017). In brief, the whole dried plant of dandelion was extracted with 50% ethanol three times, concentrated under vacuum, and then purified by macroporous resin column chromatography. The water eluent was discarded, and the 30% ethanol eluent portion was collected, evaporated, and sprayed to dryness. In cell experiments, dandelion extract was dissolved in 50% DMSO to prepare a stock solution and diluted with the culture medium. The final concentration of DMSO is 0.5%. UHPLC-ESI-Orbitrap MS/MS analysis results showed 20 compounds were identified in dandelion extract and most of them are flavonoids and phenolic acids (Supplementary Figure S1) (Li et al., 2017).

### Transfection of CHKA siRNA

MDA-MB-231 cells at a density of 1.5×10^5^ were transfected withCHKA siRNA (5′- CAU​GCU​GUU​CCA​GUG​CUC​C-3′) (RiboBio Co., Ltd. Guangzhou, China) using Lipofectamine 2000. The scrambled siRNAwas used as the negative control. The final concentration of CHKAsiRNA and scramble siRNA is 50 nM. The transfected cells withscramble or CHKA siRNA were cultured for 24 h and then assessed using Western blotting.

### MTT assay

MTT assay was performed to measure the cell viability. Briefly, MCF-10A, MDA-MB-231, and MDA-MB-468 cells were plated in 96-well plates (7×10^3^ cells/well) and cultured overnight, and then treated with dandelion extract (0, 10, 20, 40, 80, and 160 μg/ml) in DMEM with 1% FBS for 24 h. After washing with 1×PBS, cells were incubated with 0.5 mg/ml MTT solution (100 μl per well) at 37°C for 4 h. Afterward, the supernatant was discarded, and 100 μl of DMSO was added to dissolve the formazan crystals. The optical density was determined at 570 nm using Tecan Infinite 200 pro. The data was analyzed and visualized by GraphPad Prism 9.0.

### Wound-healing assay

The cell migration was performed using a wound-healing assay. MDA-MB-231 and MDA-MB-468 cells were plated in 6-well plates at 4 × 10^5^ cells per well and incubated to grow for 90% confluence. Afterward, the yellow pipette tips were used to make linear scratches, and the cell debris was removed by washing with 1×PBS. Then the cells were cultured in DMEM with 1% FBS and treated with dandelion extract at different concentrations (0, 10, 20, 40, 80, and 160 μg/ml). The images were captured by Leica DMi8 inverted microscope. The scratch width was measured by ImageJ software, and the rate of wound closure was calculated and analyzed by GraphPad Prism 9.0.

### Animal model construction

To evaluate the antitumor effects of dandelion extract *in vivo*, we constructed the MDA-MB-231 xenograft model and the 4T1 breast cancer model using NOG mice and BALB/C mice, respectively. Female NOG mice and BALB/C mice (6–8 weeks old) were purchased from Beijing HFK Bioscience Co. Ltd. (Beijing, China) and housed in a specific pathogen-free environment with a 12-h light/dark cycle and adequate food and water. All procedures involving mice were followed by the animal ethics guidelines and were approved by the Peking University Animal Research Committee. Briefly, after a 1-week acclimation, 2.5 × 10^6^MDA-MB-231 cells (resuspended in 100 μl Matrigel to NOG mice) and 2 × 10^4^ 4T1 cells (resuspended in 100 μl PBS to BALB/C mice) were implanted into the fourth inguinal mammary gland. When the tumor was palpable, the mice were randomized into three groups (n = 6 in the MDA-MB-231 xenograft model per group, n = 8 in the 4T1 model per group), including control (intraperitoneal injection with 0.1%sodium carboxymethylcellulose) and dandelion extract group (intraperitoneal injection with 50 and 100 mg/kg dandelion extract dissolved 0.1%sodium carboxymethylcellulose). Tumor volume was measured every 2–3 days and calculated by the following formula: tumor volume (mm^3^) = 0.5 × d_1_ × d_2_^2^, where d_1_ is the longest diameter and d_2_ is the shortest diameter. At the end point of animal experiments (depending on the tumor sizes), the resected tumors were weighed, and the plasma was harvested.

### Network pharmacology analysis

The chemical ingredients from dandelion extract were identified by liquid chromatography-mass spectrometry in our previous study (Li et al., 2017). Their chemical structures and SMILES ID were obtained from the PubChem database (Kim et al., 2019). The corresponding putative targets of the chemical components were predicted by three different databases, including SwissTargetPrediction (Daina et al., 2019), SEA (Keiser et al., 2007), and PharmMapper (Wang et al., 2017). TNBC-associated targets were collected from DisGeNET (Piñero et al., 2017) and GeneCards (Stelzer et al., 2016) databases with the keyword“triple-negative breast cancer”. To evaluate the reliability of our method, we validated the precision of the predicted targets based on recall via the means of literature mining. The precision rate is calculated by (the number of the intersection of the predicted targets and reported biomolecules)/(the number of predicted targets) × 100%. The overlapping genes between the chemical components of dandelion extract and TNBC were virtualized by the Venn diagram. Notably, only human genes were retained. The “compound-disease-targets” network and its topological analysis were performed via Cytoscape software (version 3.6.1). Gene Ontology (GO) functional enrichment and Kyoto Encyclopedia of Genes and Genomes (KEGG) pathway enrichment were performed using the DAVID database. Their results were visualized by the ggplots package in R software.

### Multi-omics analysis

In this study, we performed quantitative proteomics, untargeted metabolomics, and untargeted lipidomic analysis on cell samples to reveal the metabolic vulnerabilities of TNBC after dandelion extract treatment. Three batches of MDA-MB-231 cell samples with equal treatment were used for multi-omics analysis. And the tumor tissue and plasma from the 4T1 mice model were used for the lipidomics. Briefly, MDA-MB-231 cells were divided into two groups that were treated with vehicle (50% DMSO) or dandelion extract (40 μg/ml) with a sublethal concentration (40 μg/ml) based on its IC50 value, respectively. The cells were cultured in DMEM supplemented with 1% FBS (triplicate for each group) for 24 h. Then, the cell samples were washed with 1×PBS and collected into different centrifuge tubes for LC-MS/MS analysis. The differentially expressed proteins were obtained by Tandem Mass Tag (TMT) labeling quantitative proteomics via HPLC-MS/MS. And the significantly changed metabolites were identified by untargeted metabolomics and lipidomic via UPLC-MS/MS. The specific conditions and processes of multi-omics were shown in Supplementary Materials.

### Quantitative real-time polymerase chain reaction

Total RNA was extracted from cells using Trizol reagent (Invitrogen, United States), and its concentration was measured via NanoDrop 2000 (Thermo, United States). cDNAs were synthesized using a Hifair II 1st Strand cDNA Synthesis Kit (Yeasen Biotech, China). qRT-PCR was performed using SYBR Green qPCR Supermix (Applied Biosystems, United States) on 7,500 Fast Real-Time PCR Systems (Thermo, United States). The primers were from Sangon Biotech (Shanghai, China), and their sequences are listed in Supplementary Table S1. The qRT-PCR procedure for quantitative amplification was 95°C for 5 min, followed by 40 cycles of 15 s at 95°C, the 20 s at 60°C, and 40 s at 72°C. The data was analyzed and visualized via GraphPad 9.0.

### Western blotting

Western blotting was performed to confirm the results of the integrated analysis. Briefly, protein concentrations were determined by a BCA Kit (Thermo, United States). Protein samples (20 μg) were separated by SDS-PAGE and transferred onto PVDF membrane (Millipore, United States). Then the membranes were blocked with 5% skim milk in TBS solution supplemented with 0.1% Tween-20 (TBST) for 1 h at room temperature and subsequently incubated overnight at 4°C with specific primary antibodies. After that, membranes were washed with TBST and incubated with horseradish peroxidase-conjugated secondary antibodies at room temperature for 1 h. Finally, the protein bands were visualized by an enhanced chemiluminescence reagent kit (Millipore, MA). The specific information of antibodies used in this study is shown in Supplementary Table S2. Densitometry was quantitated using ImageJ software and normalized to GAPDH expression.

### Molecular modeling analysis

The molecular structures of compounds were obtained from the PubChem database, and the protein crystal structure of the candidate target was obtained from the RCSB Protein Data Bank (PDB) database. The protein structures were processed via AutoDockTools to remove ligand and water molecules, compute Gasteiger charges, add polar hydrogens, and merge non-polar hydrogens. The prepared protein structures and compounds were saved in PDBQT format. The binding box was set to contain all protein 3D structures, and other docking parameters followed the default value in AutoDock Vina. Next, the structure potential energy diagram and hydrogen bonds were shown in PyMOL.24.

### Statistical analysis

Data were shown as the mean ± standard error (SD) from three independent experiments. Evaluation of the data was performed by a two-tailed Student’s t-test. *p < 0.05* was considered a statistically significant difference.

## Results

### Dandelion extract inhibited TNBC cell proliferation and migration *in vitro* and *in vivo*

**FIGURE 2:**
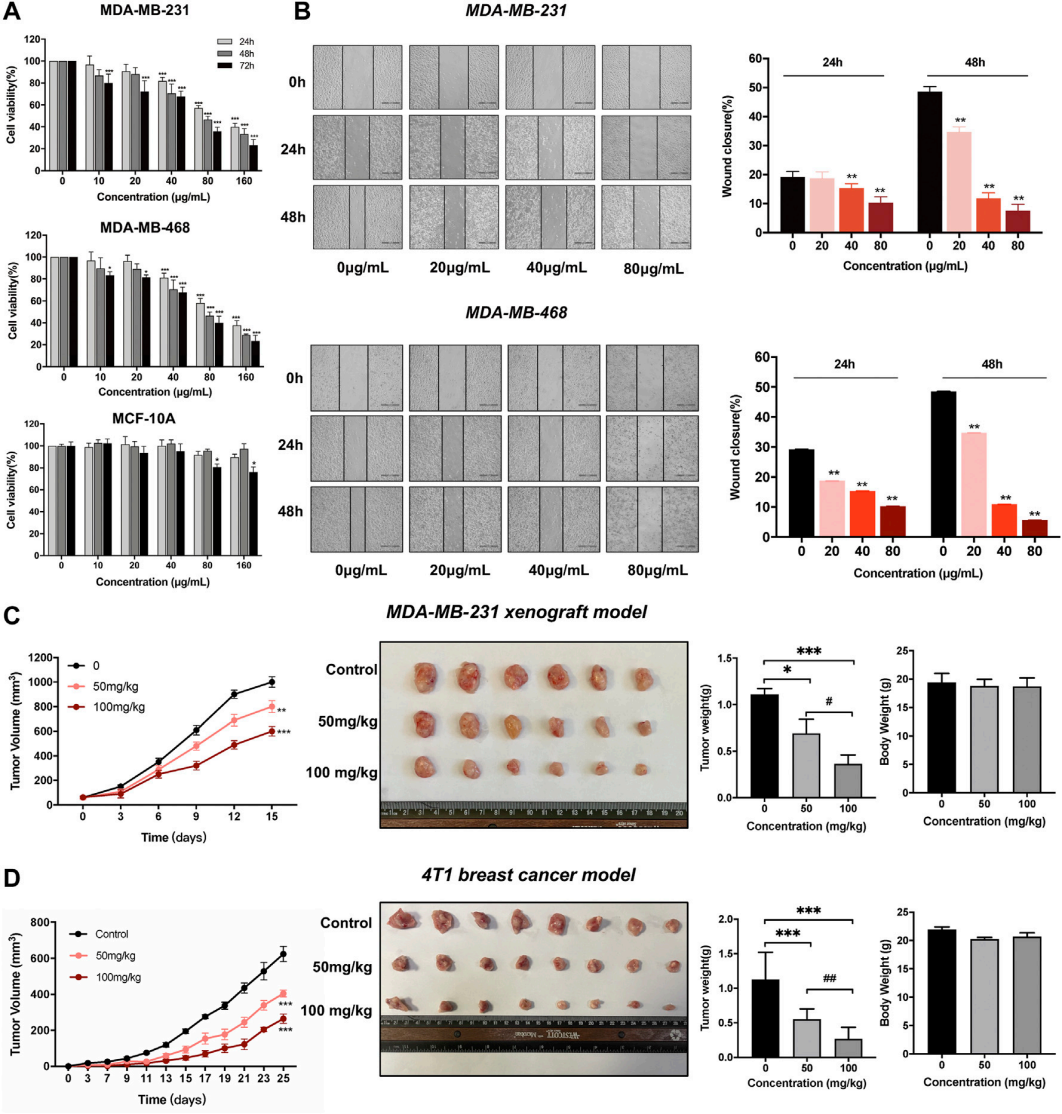
Dandelion extract inhibited TNBC cells malignant phenotypes and tumor growth in breast cancer mice model. **(A)** Dandelion extract inhibited cell proliferation on MDA-MB-231, MDA-MB-468, and MCF-10A. *p < 0.05, **p < 0.01, ***p < 0.005 vs. 24 h. **(B)** Dandelion extract inhibited TNBC cell migration in a dose-dependent manner. *p < 0.05, **p < 0.01, ***p < 0.005 vs. 0 μg/ml **(C)**Dandelion extract reduced the tumor growth in MDA-MB-231 xenograft model. **(D)** Dandelion extract reduced the tumor growth in 4T1BALB/C mice model. *p < 0.05, **p < 0.01, ***p < 0.001vs. control group (0 mg/kg), ^*#*^ *p < 0.05*, ^*##*^ *p < 0.01*, ^*###*^ *p < 0.001* vs. dandelion extract (50 mg/kg).

In this study, we performed the MTT and wound-healing assay on MCF-10A, MDA-MB-231, and MDA-MB-468 cells to assess the antitumor effects of dandelion extract. As shown in Figure 2A, TNBC cell viability was reduced in a dose-and time-dependent manner after dandelion extract treatment. The IC_50_ values of dandelion extract on MDA-MB-231 and MDA-MB-468 were 110.8 ± 9.2 μg/ml and 107.9 ± 5.6 μg/ml for 24 h, respectively. However, dandelion extract only showed its inhibitory effect on MCF-10A at the highest concentration (160 μg/ml) at 48 and 72 h. Moreover, the wound closure ability of TNBC cells was significantly decreased by dandelion extract in a dose- and time-dependent manner (Figure 2B). These results suggested that dandelion extract could preferably inhibit proliferation and migration in TNBC cells rather than normal breast cells. To further determine whether dandelion extract could suppress tumor growth in the MDA-MB-231 xenograft model and 4T1 mice model, mice were given dandelion extract (50 and 100 mg/kg) daily for15 and 20 days, respectively. We found that dandelion extract could significantly reduce the tumor volume and weight in a dose-dependent manner in the MDA-MB-231 xenograft model and 4T1 mice model. Notably, there were no obvious differences in body weights after dandelion extract treatment (Figures 2C,D). These results indicated that dandelion has potent inhibitory effects against TNBC *in vivo* and *in vitro*.

### Network pharmacology speculated the possible regulatory mechanisms of dandelion extract against TNBC

**FIGURE 3:**
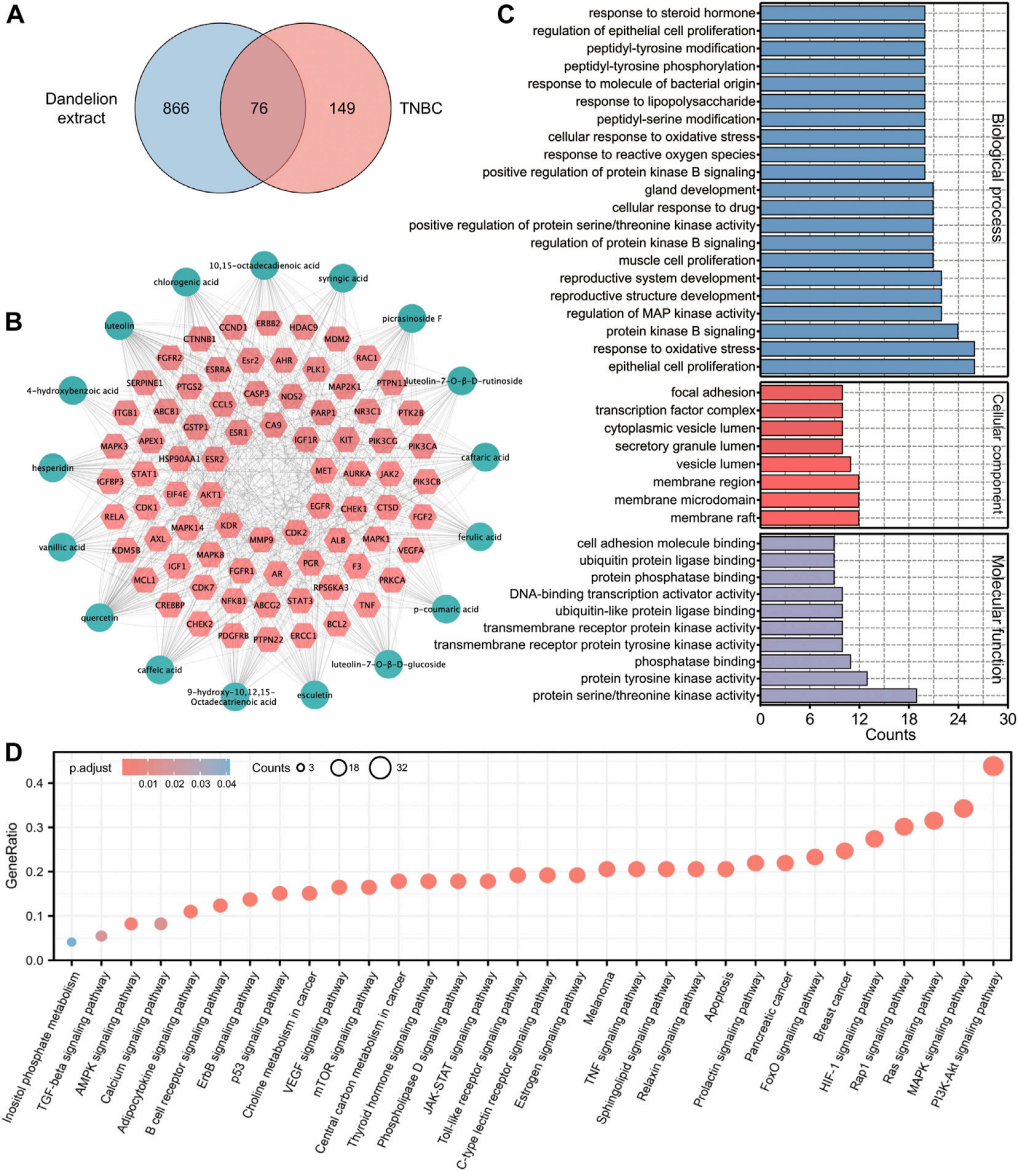
Network pharmacology analysis predicted the underlying regulatory mechanisms of dandelion extract against TNBC. **(A)** Venny diagram of the overlapping targets of dandelion extract and TNBC. **(B)** Compounds-disease-targets network. The pink diamond indicated the overlapping genes and the light green circles indicated the compounds in dandelion extract. **(C)** GO analysis for the overlapping genes. The *x*-axis represents the gene counts, and the *y*-axis represents the enriched terms. (*p < 0.05*). **(D)** KEGG pathway enrichment for the overlapping genes. The *x*-axis represents the enriched terms, and the *y*-axis represents the gene ratio (*p < 0.05*).

Network pharmacology could exhibit the complexities among compounds, diseases, and biological systems from a network perspective and thus predict their interacted mechanisms (Zhang et al., 2019). Hence, the network pharmacology analysis was used to conjecture the potential mechanisms of dandelion extract against TNBC and screen for the main bioactive compounds in dandelion extract. Our previous studies identified 22 bioactive compounds from dandelion extract via LC-MS, among which 17 compounds obtained their SMILE IDs from the PubChem database to perform network pharmacology analysis. The chemical structures and detailed information are shown in Supplementary Figures S1, S2, and Supplementary Table S3, respectively. Finally, a total of 942 compound-related putative targets and 225 TNBC-associated targets were collected after discarding the replicate, and 76 overlapped targets were merged (Figure 3A). All targets were put in the UniProt database to obtain their standard gene synonyms (Supplementary Table S4), and the precision rates of predicted targets were high.

Subsequently, we constructed a “compounds-disease-targets” network comprised of 11 nodes and 22 edges and analyzed its topological parameters, including degree, betweenness centrality, and closeness centrality (Figure 3B). The parameter values could reflect the importance of each compound in the network. Table 1 showed eight compounds with parameter values greater than the average, like luteolin, quercetin, and 10,15-octadecadienoic acid, which could be considered the major bioactive components in dandelion extract. The detailed parameters information was displayed in Supplementary Table S5. Then, GO functional analysis and KEGG pathway enrichment were used to speculate the possible regulatory pathways of dandelion extract against TNBC. Figure 3C showed multiple enriched terms, including cell proliferation, oxidative stress, membrane microdomain, and protein tyrosine kinase activity, suggesting that dandelion extract might function its anti-TNBC effects by acting on these biological processes. Moreover, as shown in Figure 3D, multiple signaling pathways involved in tumorigeneses and tumor progression were enriched, and the PI3K/AKT signaling pathway ranked first, indicating that it might play an important role in the inhibitory effects of dandelion extract against TNBC. Besides, metabolic-associated pathways, such as the AMPK signaling pathway and the choline metabolism in cancer, suggested the possible lipid regulatory effects of dandelion extract on TNBC. In general, the network pharmacology provided a systematic predictive analysis of the regulatory mechanisms of dandelion extract against TNBC *in silico*.

**TABLE1:** The topological parameters of compounds from the dandelion extract ranked Top 8.

Ranking	Compounds	Degree	Betweenness centrality	Closeness centrality
1	luteolin	44	0.07947041	0.53142857
2	quercetin	43	0.07417193	0.52542373
3	10,15-octadecadienoic acid	41	0.0716824	0.51381215
4	9-hydroxy-10,12,15- octadecatrienoic acid	40	0.10063292	0.50819672
5	caffeic acid	39	0.07271655	0.5027027
6	ferulic acid	39	0.08650739	0.5027027
7	picrasinoside F	38	0.07415693	0.4973262
8	vanillic acid	36	0.13189874	0.48691099

### Proteome and metabolome profiles of MDA-MB-231 cells after dandelion extract treatment

In order to further explore the regulatory mechanisms of dandelion extract against TNBC, TMT-based quantitative proteomics and untargeted metabolomics were performed on cell samples. The unsupervised principal component analysis (PCA) showed significant differences between the two groups (Supplementary Figure S3A). As a result, a total of 5,049 proteins were identified, and 3,932 proteins were quantitated. Based on the screen criteria of fold change >1.2 and *p*-value (*p < 0.05*)*,* we finally identified144 differentially expressed proteins, including 82 upregulated proteins and 62 downregulated proteins (Supplementary Figure S3B). Then, GO function enrichment analysis indicated that the differentially expressed proteins involved multiple biological functions, including metabolic processes (Supplementary Figure S3C). Besides, by using clusters of orthologous groups (COG) annotation, differentially expressed proteins could be divided into three different functional categories involved in metabolism, cellular process and signaling, and information storage and processing, accounting for 24.6, 19.3, and 43.0%, respectively (Supplementary Figure S3D).

As for metabolome profiles, the cell samples from two groups could be distinguished well by the PCA and OSPL-DA (Supplementary Figures S3E,F). Totally, based on the screen criteria with VIP ≥1, fold change≥ 2, and *p*-value (*p < 0.05*), we determined 33 significantly changed metabolites (28 upregulated and 5 downregulated) that belonged to 9 categories, including glycerophospholipids, amino acids, carboxylic acids, heterocyclic compounds, coenzyme and vitamins, etc. (Supplementary Figure S3G). Notably, glycerophospholipids spices were the majority metabolites that accounted for 54.5%. The results of proteomics and untargeted metabolomics suggested that dandelion extract exerted its antitumor effects on TNBC by primarily interfering with the lipid metabolic processes, especially in glycerophospholipid metabolism.

### Lipidome profiling of MDA-MB-231 after dandelion extract treatment revealed its primary metabolic regulatory processes

**FIGURE 4:**
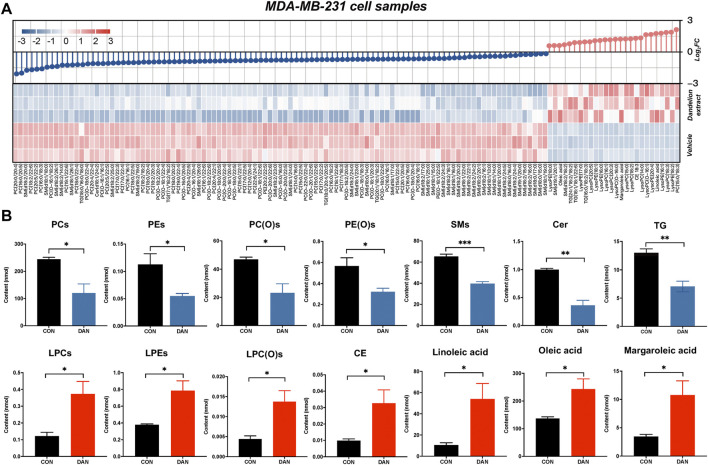
The lipidomics profiles of MDA-MB-231 cells after dandelion extract treatment. **(A)** The heatmap and change dumbbell charts of the significantly changed metabolites of untargeted lipidomics. **(B)** The changes of the significantly differential metabolites of untargeted lipidomics.

To further address the lipid regulatory mechanisms of dandelion extract on TNBC, untargeted lipidomic was performed using TNBC cell, tumor tissue and plasma samples to find out more significantly changed lipid metabolites and their related metabolic pathways. The MDA-MB-231 cell samples treatment was consistent with proteomics and untargeted metabolomics, and the tumor tissue and plasma samples were obtained from the mice model. The unsupervised PCA and OSPL-DA indicated that all the samples from the two groups were significantly separated (Supplementary Figures S4–S6). The significantly changed lipid metabolites were identified by the screen criteria with VIP ≥1 and *p-*value (*p < 0.05*). Finally, we obtained 1,331 significantly changed lipid metabolites (26 upregulated and 105 downregulated) in cell lipidomics that could be classified into glycerophospholipids, sphingolipids, cholesterol, and unsaturated fatty acids (Figure 4A). The primarily disrupted lipids were glycerophospholipids, including phosphatidylcholine (PC), phosphatidylethanolamine (PE), lysophosphatidylcholine (LPC), alkyl phosphatidylcholine, alkyl phosphatidylethanolamine, and lysophosphatidylethanolamine (LPE). The total relative abundance of each lipid species was visualized in Figure 4B. Specifically, the contents of PC, PE, alkyl phosphatidylcholine, alkyl phosphatidylethanolamine, triglyceride (TG), sphingomyelin (SM), and ceramide (Cer) were significantly decreased after dandelion extract treatment. Inversely, the total relative levels of LPC, LPE, cholesteryl ester (CE), linoleic acid (LA), oleic acid (OA), and margaroleic acid were increased.

**FIGURE 5:**
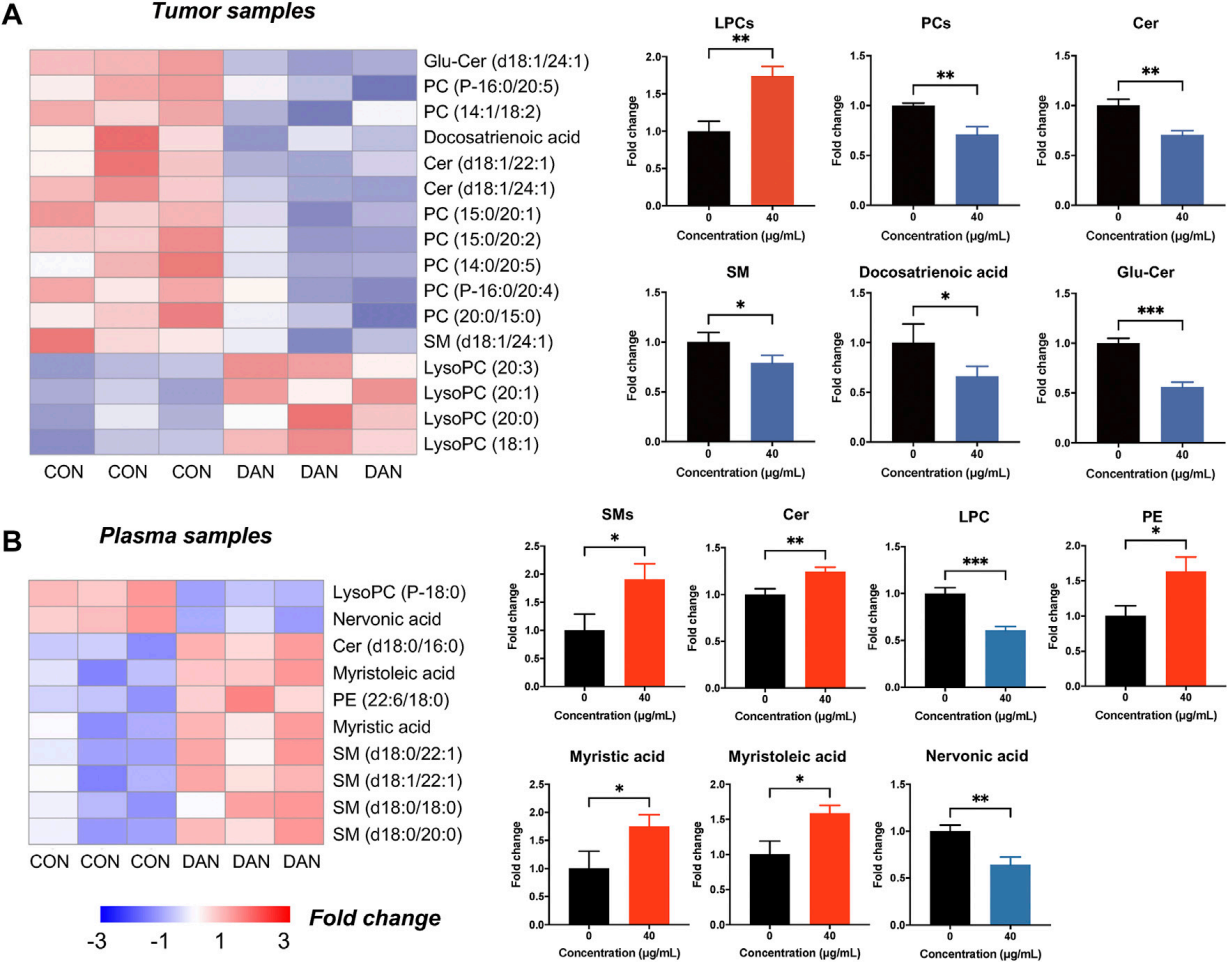
The lipidomics profiles of TNBC tumor tissue and plasma after dandelion extract treatment. **(A)** The heatmap charts of the significantly changed metabolites in tumor tissue lipidomics. **(B)** The heatmap charts of the significantly changed metabolites in plasma lipidomics.

Furthermore, a total of 16 lipid metabolites were changed significantly in tumor tissue lipidomics (12 upregulated and 4 downregulated). PC, SM, Cer, and docosatrienoic acid were decreased, and LPC was increased in tumor tissues (Figure 5A). Notably, the variation trends of PC, LPC, SM, and Cer in tumor tissue were consistent with the cell lipidomics, indicating that dandelion extract interfered with the synthesis of phospholipids and sphingolipids of TNBC both *in vitro* and *in vivo*. Besides, a total of 10 significantly changed lipid metabolites were identified by plasma lipidomics (8 upregulated and 2 downregulated) (Figure 5B). Sphingolipid metabolites such as SM and Cer, PE, and the unsaturated fatty acids like myristoleic acid and myristic acid were increased in plasma. LPC and nervonic acid were decreased in plasma. In addition, the KEGG pathway enrichment indicated glycerophospholipid metabolism, sphingolipid metabolism, and unsaturated fatty acid metabolism were the primary regulatory processes (Supplementary Figure S7).

### Integrated analysis identified the primary metabolic pathways and biomarkers in the regulation of dandelion extract on TNBC

**FIGURE 6:**
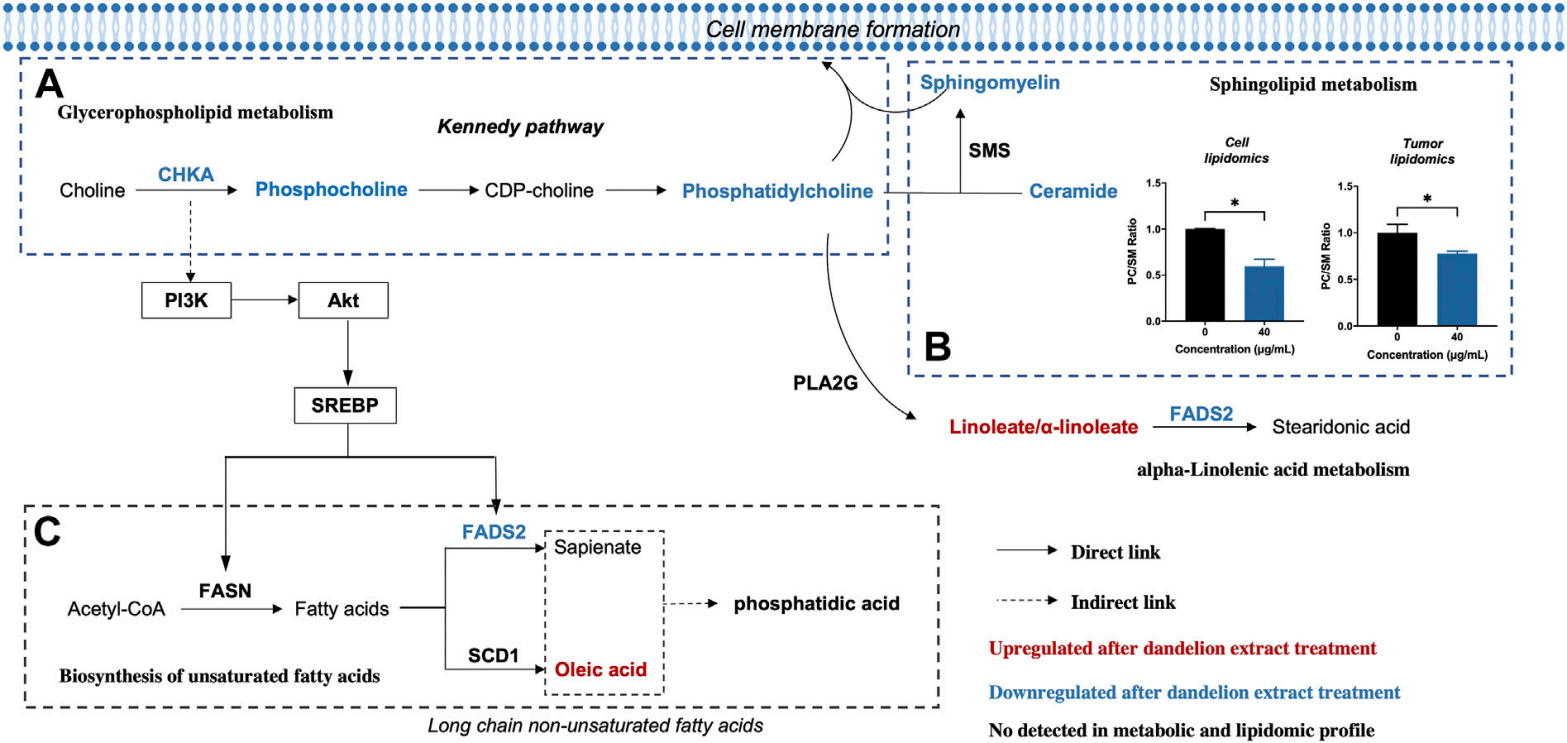
Altered metabolic pathways of MDA-MB-231 cell after dandelion extract treatment. **(A)** The Glycerophospholipid metabolism. **(B)** The Sphingolipid metabolism. **(C)** The biosynthesis of unsaturated fatty acids.

Afterward, we performed an integrated analysis based on the results of multi-omics and network pharmacology to find out the co-regulated lipid metabolic pathways and identified the primary regulatory processes and biomarkers in the regulation of dandelion extract against TNBC. As shown in Figure 6, the co-regulated pathways included linoleic acid metabolism (map02591), glycerophospholipid metabolism (map00564), biosynthesis of unsaturated fatty acids (map01040), alpha-linolenic acid metabolism (map00592), and sphingolipid metabolism (map00600). By matching the changed metabolic-associated proteins identified in proteomics, we found that choline kinase α (CHKA) and FADS2 were highly connected with the metabolites in glycerophospholipid metabolism and the biosynthesis of unsaturated fatty acids, respectively. In the glycerophospholipid metabolism, CHKA expression was decreased, and the levels of its downstream metabolites PCho, PC, and PE were decreased simultaneously (Figure 6A). Besides, the content levels of SM and Cer were also reduced. Notably, both SM and PC are important components of cell membranes, and the PC/SM ratio could reflect the fluidity of cell membranes (Mao et al., 2022), which is closely related to the invasive and migrative capacities of tumor cells (Li et al., 2021a). Our results showed that the PC/SM ratio was reduced both in the TNBC cells and tissues (Figure 6B) and demonstrated that dandelion extract could impede cell membrane formation and reduce the cell membrane fluidity via disrupting the biosynthesis of PC and SM, ultimately leading the inhibition of TNBC cell growth and migration.

On the other hand, in the biosynthesis of unsaturated fatty acids, the protein expression levels of FADS2 were decreased, and meanwhile, linoleic acid and oleic acid were increased (Figure 6C). Therefore, CHKA and FADS2 were considered the critical biomarkers that accounted for lipids regulatory effects of dandelion extract against TNBC. Based on the KEGG enrichment in network pharmacology, CHKA might interact with the PI3K/AKT pathway, which plays a vital role in tumor growth, proliferation, and metabolism (Alzahrani, 2019). Besides, SREBP and FADS2 were the downstream molecules of the PI3K/AKT pathway (Griffiths et al., 2013; Zhang et al., 2020). Next, we would perform experimental validations on the above molecules.

### Dandelion extract disrupted metabolic-associated pathways *in vitro*

**FIGURE 7:**
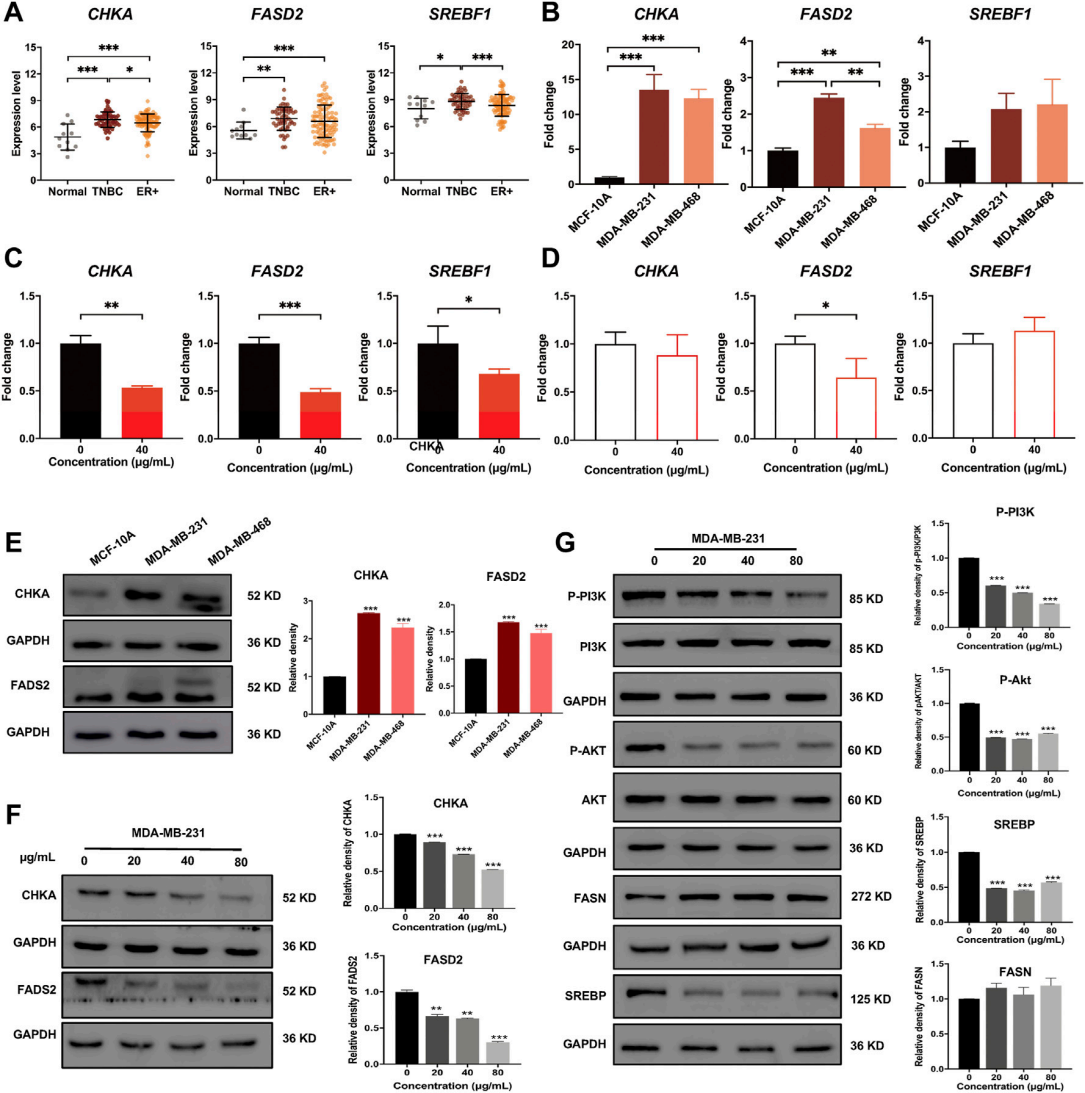
Dandelion extract inhibited mRNA and protein expression levels of metabolic-associated proteins and pathways. **(A)** The mRNA expression levels of *CHKA, FADS2*, and*SREBF1* in normal, TNBC, and ER^+^ breast cancer tissues based on the GSE65194 dataset. **(B)** The mRNA expression levels of *CHKA, FADS2*, and*SREBF1* in TNBC and normal cells by RT-PCR. **(C)** The mRNA expression levels of *CHKA, FADS2*, and*SREBF1* after dandelion extract treatment in MDA-MB-231 cells by RT-PCR. **(D)** The mRNA expression levels of *CHKA, FADS2*, and*SREBF1* after dandelion extract treatment in MCF-10A cells by RT-PCR. **(E)** The protein expression levels of CHKA and FADS2 in MCF-10A and TNBC cells. **(F)** The protein expression levels of CHKA and FADS2 in MDA-MB-231 cells after dandelion extract treatment. **(G)** The expression levels of p-PI3K, p-AKT, SREBP, and FASN in MDA-MB-231 cells after dandelion extract treatment. *p < 0.05, **p < 0.01, ***p < 0.001.

To further demonstrate the accuracy of the integrated analysis results, we performed experimental validations on the identified biomarkers CHKA and FADS2, as well as their related regulatory pathways. First, we compared the mRNA expression levels based on a transcriptome profile with130 breast cancer samples (41 TNBC and 89 ER^+^ BC) and 11 normal breast tissue samples (GSE65194 dataset from Gene Expression Omnibus database). These data indicated that mRNA expression levels of *CHKA*, *FADS2*, and *SREBF1*werehigher in breast cancer samples than in normal breast tissue, and their levels in TNBC were significantly changed compared to ER^+^ breast cancer samples (Figure 7A). Then, qRT-PCR results confirmed that the mRNA levels of *CHKA* and *FADS2* were higher in TNBC cells than in MCF-10A, but there were no significant differences in *SREBF1* mRNA levels (Figure 7B). After dandelion extract treatment, the mRNA expression levels of *CHKA, FADS2*, and *SREBF1*in MDA-MB-231 were both decreased (Figure 7C). For MCF-10A cells, dandelion extract only reduced the mRNA level of *FADS2*but with no significant effect on the *CHKA* and *SREBF1*(Figure 7D)*.*

Subsequently, western blot results showed that the expression levels of CHKA and FADS2 in TNBC cells were higher than in MCF-10A (Figure 7E). After dandelion extract treatment, the expression of CHKA and FADS2 were reduced in a dose-dependent manner in TNBC cells (Figure 7F). Moreover, the phosphorylation levels of PI3K and AKT were also reduced, suggesting that the PI3K/AKT pathway was inhibited by dandelion extract (Figure 7G). And the expression of SREBP, a downstream target of the PI3K/AKT pathway, was reduced accordingly. It is demonstrated that FASN and FADS2 were the targets of SREBP, accounting for the fatty acid synthesis and desaturation, respectively (Griffiths et al., 2013). However, our results showed that dandelion extract could inhibit the expression of SREBP and FADS2, but did not have apparent inhibitory effects on FASN (Figure 7G). The above results confirmed that dandelion extract could reduce the mRNA and protein expression levels of CHKA and FADS2 and inhibit the PI3K/AKT pathway in TNBC cells.

**FIGURE 8:**
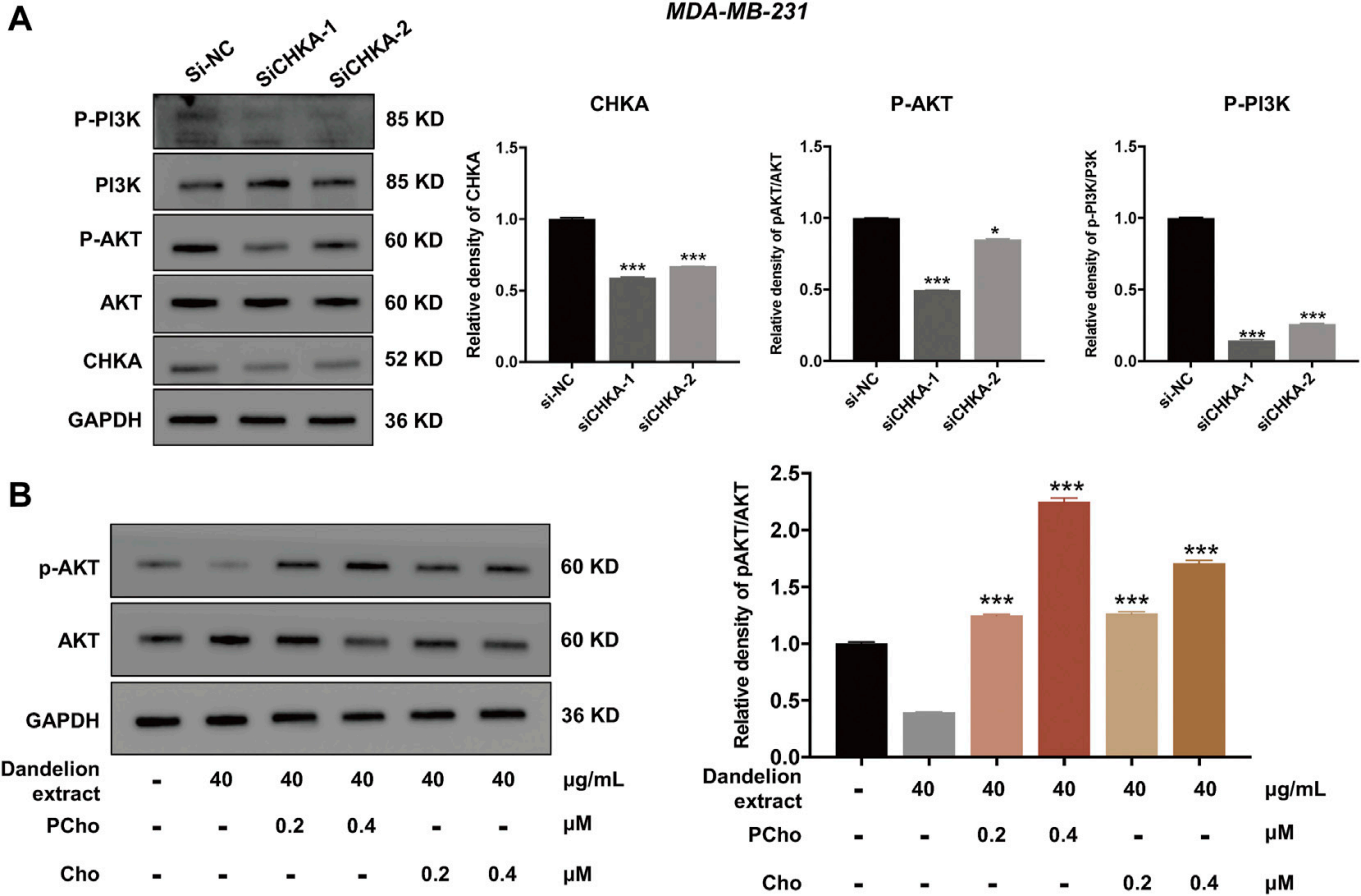
Dandelion extract inhibited mRNA and protein expression levels of metabolic-associated proteins and pathways. **(A)** Inhibition of CHKA expression decreased the phosphorylation levels of PI3K and AKT. *p < 0.05, **p < 0.01, ***p < 0.001 vs. si-NC. **(B)**The exogenous addition of Cho and PCho reversed the decreased phosphorylation levels of AKT in MDA-MB-231 cells. *p < 0.05, **p < 0.01, ***p < 0.001 vs. dandelion extract (40 μg/kg).

Furthermore, our previous results showed that the content of PCho was decreased due to the inhibition of CHKA by dandelion extract. It is reported that PCho, the direct metabolite of CHKA, is abnormally elevated in various cancers and is able to provide phosphate molecules for AKT activation, thus promoting tumor proliferation (Venkatesh et al., 2012). To explore the interaction between CHKA and PI3K/ATK signaling pathway, MDA-MB-231 cells were transfection with CHKA siRNA. Western blot results showed CHKA expression in MDA-MB-231 cells was significantly inhibited by CHKA siRNA, and the phosphorylation levels of PI3K and AKT were similarly reduced after endogenous inhibition of CHKA expression (Figure 8A). In contrast, exogenous addition of Cho and PCho could reverse the decreased AKT phosphorylation levels in MDA-MB-231 cells by dandelion extract (Figure 8B). The above results suggest that dandelion extract could inhibit the PI3K/AKT signaling pathway by reducing the CHKA expression and reducing the synthesis of Cho into PCho, thereby reducing the phosphorylation level of AKT.

### Luteolin might be the primary bioactive compound in dandelion extract via inhibiting CHKA

**FIGURE 9:**
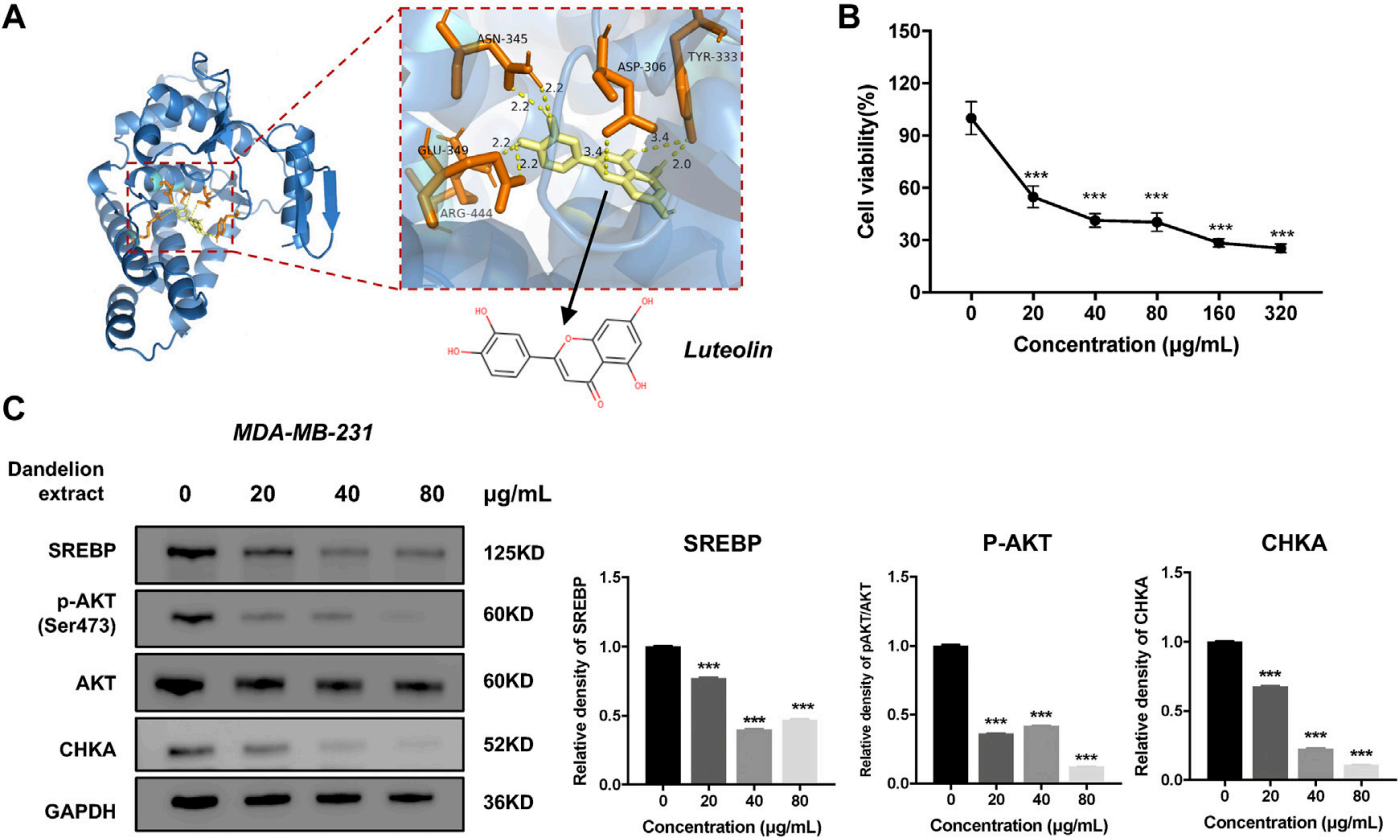
The luteolin could inhibit TNBC cell proliferation and reduce the expression level of CHKA. **(A)** The interactions of the potential crystal structure of CHKA domain in complex with luteolin based on the molecular docking simulation. The dotted line between the compound and the protein refers to the hydrogen bond. TYR, tyrosine; CLU, glutamic acid; SER, serine; LEU, leucine; ASN, Asparagine; ASP, Aspartic acid; ARG, Arginine.**(B)**. Luteolin inhibited MDA-MB-231 cell proliferation by MTT assays. **(C)**. Luteolin reduced the expression levels of SREBP, CHKA, and p-AKT. *p < 0.05, **p < 0.01, ***p < 0.001 vs. 0 μg/ml.

Integrated analysis and experimental validation revealed that CHKA played a vital role in the inhibition of dandelion extract on TNBC. And the network pharmacology analysis speculated eight compounds that were essential for dandelion’s multi-pharmacological effects. To address whether the compounds could interact with CHKA directly, molecular docking simulation was performed to calculate the binding scores between CHKA and compounds. The binding scores of CHKA and its specific inhibitors rabusertib or AZD7762 were the baseline. The molecular structures of compounds were obtained from the PubChem database. The 3D crystal structure of CHKA and hemicholinium-3 complex (PDB code: 3F2R) was derived from the PDB. Notably, hemicholinium-3 should be removed from the complex with the PyMOL, and the pure 3D crystal structure of CHKA only remained. Molecular docking was processed via AutoDock Vina, and the results were viewed and analyzed using PyMOL. The results showed that eight compounds exhibited inconsistent binding capacities with CHKA (Table 2 and Figure 9A), and picrasinoside F, luteolin, and esculetin possess the strongest binding capacity to CHKA. Subsequently, we performed the MTT assays using luteolin on the TNBC cells, and it showed a potent inhibitory effect against MDA-MB-231 cells (Figure 9B). Western blot results demonstrated that luteolin could decrease the expression levels of CHKA, SREBP, and p-AKT, which were consistent with the tendency after dandelion extract treatment (Figure 9C), suggesting it might be a novel CHKA inhibitor and the primary bioactive compound in dandelion extract.

**TABLE 2:** The binding scores between CHKA andthe compounds from dandelion extract.

Ranking	Target	Ligands	Binding score (kcal/mol)
-	CHKA	rabusertib	−7.98
-	CHKA	AZD7762	−7.32
1	CHKA	picrasinoside F	−7.5
2	CHKA	luteolin	−6.94
3	CHKA	quercetin	−5.47
4	CHKA	caffeic acid	−4.82
5	CHKA	9-hydroxy-10,12,15-octadecatrienoic acid 7	−4.44
6	CHKA	ferulic acid	−4.39
7	CHKA	vanillic acid	−4.3
8	CHKA	10,15-octadecadienoic acid	−3.73

## Discussion

Metabolic reprogramming is a prominent hallmark of cancer cells (Deberardinis and Chandel, 2016). The alterations in lipid metabolic activities provide energy and the building stocks of the cell membrane to meet the increased needs for tumor cell proliferation (Peck and Schulze, 2016). Simultaneously, it had been reported that certain Chinese herbal medicines and their components could disrupt the reprogrammed metabolic processes, enhancing the antitumor efficacies of conventional therapeutics or leading to tumor cell death directly (Zhong et al., 2016; Wang et al., 2021b). Dandelion is a classic herbal medicine in treating mammary diseases and has been demonstrated to possess antitumor and lipid regulatory effects. In the present study, dandelion extract was confirmed to inhibit the proliferation and migration of TNBC cells in a time- and dose-dependent manner, and it could significantly suppress the tumor growth in the MDA-MB-231 xenograft and 4T1 mice model. TNBC has wide reprogrammed lipid metabolisms that contribute to its malignancy (Sun et al., 2020). To address the question of whether dandelion extract possesses lipid regulatory effects on TNBC and elucidate the potential mechanisms, an integrated approach composed of network pharmacology, quantitative proteomics, untargeted metabolomics, and untargeted lipidomics was performed in this study.

Network pharmacology could analyze the molecular association between compounds and diseases and predict systematic pharmacological mechanisms in a holistic manner (Gu et al., 2013). The topological analysis of network pharmacology suggested that luteolin, quercetin, 10,15-octadecadienoic acid, and other five compounds were the main bioactive component in dandelion extract and played key roles in dandelion’s antitumor effects. Besides, the enrichment analysis of network pharmacology speculated that dandelion extract regulated various biological processes and metabolic-associated pathways of TNBC. The PI3K/AKT pathway was the most remarkable among them (Figure 3).

Next, the multiple-omics technique was used to obtain the changed protein and metabolites and reveal metabolic vulnerabilities of TNBC after dandelion extract treatment. Totally, 144 differential expressed proteins and 43 significantly changed metabolites were identified by quantitative proteomics and untargeted metabolomics, respectively. These results suggested that the primary regulatory effect of dandelion extract was involved in lipid metabolism. Therefore, the untargeted lipidomics was performed subsequently, and we obtained 131 significantly changed lipid metabolites, including glycerophospholipids, sphingolipids, cholesterol, and unsaturated fatty acids. Then, through the integrated analysis of the multi-omics results and network pharmacology, we identified five co-regulated metabolic pathways, and the glycerophospholipid metabolism, linolenic acid metabolism, and biosynthesis of unsaturated fatty acids were the most significant and considered CHKA and FADS2 as the critical targets for further experimental validation *in vitro* (Figure 6).

In glycerophospholipid metabolism, multiple metabolic-associated proteins and metabolites were changed after dandelion extract treatment. Among them, CHKA is a crucial rate-limiting enzyme that converts intracellular free choline to phosphorylcholine in the *de novo* synthesis of PC (Kennedy pathway) (Andrejeva et al., 2020). CHKA and its related metabolites, including phosphorylcholine, PC, PE, LPC, and LPE, are overexpressed in primary tumor tissues of the breast, colorectal, prostate, and lung and could be considered the prognostic and diagnostic markers (Gibellini and Smith, 2010; Grinde et al., 2014) In this study, we confirmed that the mRNA and protein levels of CHKA were higher than normal and reduced after dandelion extract treatment. More importantly, the decreased CHKA expression led to the reduction of the content of its metabolites like PCho, PC, and PE. The decreased PC reduced the SM biosynthesis accordingly. PC, PE, and SM are essential components of cell membranes, and the PC/SM ratio could reflect its fluidity, which is closely related to the invasive and migrative capacities of tumor cells. Our results demonstrated that dandelion extract could disrupt the glycerophospholipids metabolism via inhibiting CHKA expression, impeding cell membrane formation, and reducing the cell membrane fluidity, ultimately leading to the inhibition of TNBC cell growth and migration. Moreover, the content of LPC and LPE were increased inversely, and thus the ratio of PC/LPC was decreased after dandelion extract treatment. The higher content of LPC is beneficial for a better prognosis (Li et al., 2021b).

In addition to the catalytic effect, CHKA could act as a mediator in regulating cell signaling transduction and promoting tumor initiation and progression (Cheng et al., 2016). For example, the c-Src-dependent link between CHKA and EGFR promoted breast cancer cell proliferation and tumorigenesis (Miyake and Parsons, 2012). Down-regulation of CHKA expression elicited the endoplasmic reticulum stress and induced apoptosis via CHOP in breast cancer (Sanchez-Lopez et al., 2013), and it is reported that CHKA could promote glioma development via activating PI3K/AKT pathways (Zou et al., 2021). In this study, the network pharmacology analysis spectacled that CHKA may interact with the P13K/AKT pathway, which is abnormally active in various tumor cells and is closely related to tumor growth, proliferation, and metabolism (Hoxhaj and Manning, 2020). The western blot results showed that the phosphorylation levels of PI3K and AKT were reduced after dandelion extract treatment, which is according to the decrease in CHKA expression. Besides, the phosphorylation levels of PI3K and AKT were similarly reduced after endogenous inhibition of CHKA expression. It is reported that PCho, the direct metabolite of CHKA, is abnormally elevated in various cancers and is able to provide phosphate molecules for AKT activation, thus promoting tumor proliferation. The content of PCho was decreased due to the inhibition of CHKA by dandelion extract based on the metabolomics results. Then, we found that the exogenous addition of Cho and PCho could reverse the decreased AKT phosphorylation levels by inhibiting CHKA in MDA-MB-231 cells. The above results indicated that dandelion extract could inhibit the PI3K/AKT signaling pathway by reducing the CHKA expression and the biosynthesis of PCho, ultimately decreasing the phosphorylation level of AKT. The exogenous addition of Cho and PCho could reverse the phosphorylation levels of PI3K and AKT, thus suggesting that the antitumor effects of dandelion extract are partially mediated by the CHKA/PI3K/AKT pathway.

**FIGURE 10:**
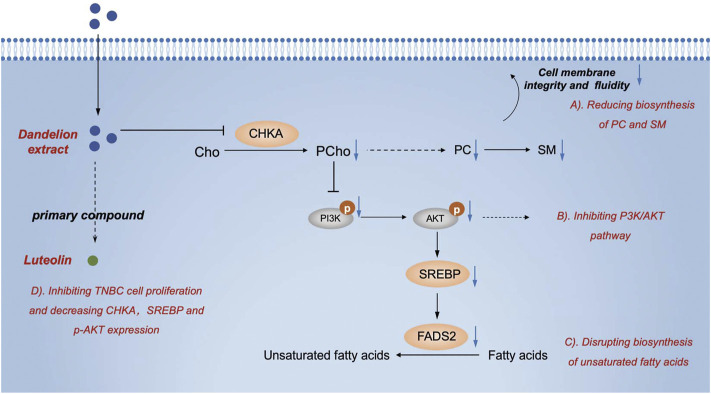
The graphic abstract of the lipid regulatory effects of dandelion extract against TNBC.

On the other hand, we found that dandelion extract could disrupt the unsaturated fatty acids metabolism, enhancing the content of oleic acid and linoleic acids but decreasing FADS2 expression. The unsaturated fatty acids are the integral macromolecules for phospholipid synthesis and cell membrane formation, and inhibition of fatty acids synthesis and desaturation is detrimental to tumor cell survival and contributes to antitumor properties (Saati and Archer, 2011; Peck et al., 2016). Some cancers rely on two desaturation pathways mediated by stearoyl-coenzyme A desaturase (SCD) and FADS2 to generate unsaturated fatty acids (Vriens et al., 2019). FADS2 is regulated by SREBP and generates unusual fatty acid sapienate, which could replace palmitoleate or oleate produced by SCD in cell membrane synthesis (Li et al., 2021b). Hence, only dual inhibition of SCD and FADS2 could result in tumor cell death directly. In this study, we confirmed that the mRNA and proteins expression levels of FADS2 were higher in TNBC cells and tissues than normal, suggesting that TNBC existed the abnormal FADS2-dependent fatty acids desaturation. After dandelion extract treatment, the mRNA and protein expression of FADS2 were decreased and SREBP expression was also reduced accordingly (Triki et al., 2020). However, we did not detect the changes of sapienate via metabolomics or lipidomics, and we speculated the content of sapienate would be reduced in theory, causing the compensatory increase of other unsaturated fatty acids, such as oleic acid and linoleic acids. Besides, the expression of FASN, another downstream target of SREBP, had no significant changes after dandelion extract treatment. Taken together, our results indicated that dandelion extract could decrease fatty acids desaturation via inhibiting the SREBP/FADS2 axis but has no apparent effects on fatty acids synthesis (Figure 10).

Notably, a recent study indicated that the aqueous extract of dandelion could exhibit therapeutic effects on TNBC by inhibiting CDK6 expression and regulating arginine and proline metabolism pathway based on its results of untargeted metabolomics (Qu et al., 2022). However, the dandelion extract in our study was extracted via 50% ethanol and purified by macroporous resin. Therefore, different extraction processes led to the differences in the types and contents of bioactive compounds in dandelion extract and the regulatory mechanisms of aqueous or ethanol extract of dandelion were not entirely consistent. Our results suggested that dandelion ethanol extract inhibited TNBC by interfering with glycerophospholipids and unsaturated fatty acids metabolism.

Furthermore, we performed the molecular docking simulation to screen the potential anti-cancer compounds from dandelion extract, which could exert its pharmacological effects by binding CHKA. The topological analysis of network pharmacology identified eight essential compounds and their binding scores between CHKA wound compared to the scores of CHKA-specific inhibitors to evaluate the binding capacity. The results indicated that picrasinoside F and luteolin possessed a high binding capacity with hydrogen bond and might consider novel potential CHKA inhibitors. Besides, we preliminary showed that luteolin had a potent inhibitory effect against MDA-MB-231 cells and inhibited the CHKA/AKT/SREBP axis, which might be the main bioactive compound in dandelion extract.

## Conclusion

In conclusion, we confirmed the antitumor effects of dandelion extract on TNBC *in vitro* and found that dandelion extract could suppress tumor growth in the MDA-MB-231 xenograft and 4T1 model. We also exhibited the proteomic and lipidomic characteristics of TNBC cells, tumor tissue, and plasma after dandelion extract treatment. Through integrated analysis and experimental invalidation *in vitro*. We demonstrated that dandelion extract could interfere with glycerophospholipids and unsaturated fatty acids metabolism by inhibiting CHKA/PI3K/AKT/FADS2 axis, ultimately resulting in the blockage of cell membrane formation and tumor cell death (Figure 10). Additionally, we speculated that picrasinoside F and luteolin were the main bioactive components in dandelion extract and might function their pharmacological effects against TNBC via binding CHKA *in silicon*.

## Data Availability

The original contributions presented in the study are publicly available. This data can be found here: https://www.ebi.ac.uk/pride/archive/, PXD035797.

## Abbreviations

TNBC, triple-negative breast cancer; ER, estrogen receptor; SREBP, sterol-regulatory element-binding proteins; FASN, fatty acid synthase; FADS2, fatty acid desaturase 2; LPC, lysophosphatidylcholine; AMPK, AMP-activated protein kinase; PC, phosphatidylcholine; PE, phosphatidylethanolamine; LPE, lysophosphatidylethanolamine; TG, triglyceride; SM, sphingomyelin; Cer, ceramide; CE, cholesteryl ester; LA, linoleic acid; OA, oleic acid.

## References

[B1] Alzahrani A. S. (2019). PI3K/Akt/mTOR inhibitors in cancer: At the bench and bedside. Semin. Cancer Biol. 59, 125–132. 10.1016/j.semcancer.2019.07.009 31323288

[B2] Andrejeva G. Gowan S. Lin G. Wong Te Fong A. C. L. Shamsaei E. Parkes H. G. (2020). De novo phosphatidylcholine synthesis is required for autophagosome membrane formation and maintenance during autophagy. Autophagy 16, 1044–1060. 10.1080/15548627.2019.1659608 31517566 PMC7469489

[B3] Bianchini G. Balko J. M. Mayer I. A. Sanders M. E. Gianni L. (2016). Triple-negative breast cancer: Challenges and opportunities of a heterogeneous disease. Nat. Rev. Clin. Oncol. 13, 674–690. 10.1038/nrclinonc.2016.66 27184417 PMC5461122

[B4] Burstein M. Tsimelzon A. Poage G. M. Covington K. R. Brown P. H. Fuqua S. A. W. (2015). Comprehensive genomic analysis identifies novel subtypes and targets of triple-negative breast cancer. Clin. Cancer Res. 21, 1688–1698. 10.1158/1078-0432.CCR-14-0432 25208879 PMC4362882

[B5] Cheng M. Bhujwalla Z. M. Glunde K. (2016). Targeting phospholipid metabolism in cancer. Front. Oncol. 6, 266. 10.3389/fonc.2016.00266 28083512 PMC5187387

[B6] Choi U. K. Lee O. H. Yim J. H. Cho C. W. Rhee Y. K. Lim S. I. (2010). Hypolipidemic and antioxidant effects of dandelion (*Taraxacum officinale*) root and leaf on cholesterol-fed rabbits. Int. J. Mol. Sci. 11, 67–78. 10.3390/ijms11010067 20162002 PMC2820990

[B7] Daina A. Michielin O. Zoete V. (2019). SwissTargetPrediction: Updated data and new features for efficient prediction of protein targets of small molecules. Nucleic Acids Res. 47, W357–W364. 10.1093/nar/gkz382 31106366 PMC6602486

[B8] Davaatseren M. Hur H. J. Yang H. J. Hwang J. T. Park J. H. Kim H. J. (2013). Taraxacum official (dandelion) leaf extract alleviates high-fat diet-induced nonalcoholic fatty liver. Food Chem. Toxicol. 58, 30–36. 10.1016/j.fct.2013.04.023 23603008

[B9] Deberardinis R. J. Chandel N. S. (2016). Fundamentals of cancer metabolism. Sci. Adv. 2, e1600200. 10.1126/sciadv.1600200 27386546 PMC4928883

[B10] Deng X. X. Jiao Y. N. Hao H. F. Xue D. Bai C. C. Han S. Y. (2021). Taraxacum mongolicum extract inhibited malignant phenotype of triple-negative breast cancer cells in tumor-associated macrophages microenvironment through suppressing IL-10/STAT3/PD-L1 signaling pathways. J. Ethnopharmacol. 274, 113978. 10.1016/j.jep.2021.113978 33716082

[B11] Duan X. Pan L. Deng Y. Liu Y. Han X. Fu H. (2021). Dandelion root extract affects ESCC progression via regulating multiple signal pathways. Food Funct. 12, 9486–9502. 10.1039/d1fo01093j 34476429

[B12] Eghlimi R. Shi X. Hrovat J. Xi B. Gu H. (2020). Triple negative breast cancer detection using LC-MS/MS lipidomic profiling. J. Proteome Res. 19, 2367–2378. 10.1021/acs.jproteome.0c00038 32397718

[B13] Gandhi N. Das G. M. (2019). Metabolic reprogramming in breast cancer and its therapeutic implications. Cells 8, E89. 10.3390/cells8020089 30691108 PMC6406734

[B14] Gauhar R. Hamayun M. Iqbal A. Khan S. A. Khan H. Shehzad A. (2017). Effect of methanolic extract of dandelion roots on cancer cell lines and AMP-activated protein kinase pathway. Front. Pharmacol. 8, 875. 10.3389/fphar.2017.00875 29234282 PMC5712354

[B15] Gibellini F. Smith T. K. (2010). The Kennedy pathway--De novo synthesis of phosphatidylethanolamine and phosphatidylcholine. IUBMB Life 62, 414–428. 10.1002/iub.337 20503434

[B16] Gong Y. Ji P. Yang Y. S. Xie S. Yu T. J. Xiao Y. (2021). Metabolic-pathway-based subtyping of triple-negative breast cancer reveals potential therapeutic targets. Cell Metab. 33, 51–64.e9. 10.1016/j.cmet.2020.10.012 33181091

[B17] Griffiths B. Lewis C. A. Bensaad K. Ros S. Zhang Q. Ferber E. C. (2013). Sterol regulatory element binding protein-dependent regulation of lipid synthesis supports cell survival and tumor growth. Cancer Metab. 1, 3. 10.1186/2049-3002-1-3 24280005 PMC3835903

[B18] Grinde M. T. Skrbo N. Moestue S. A. Rodland E. A. Borgan E. Kristian A. (2014). Interplay of choline metabolites and genes in patient-derived breast cancer xenografts. Breast Cancer Res. 16, R5. 10.1186/bcr3597 24447408 PMC3978476

[B19] Gu J. Gui Y. Chen L. Yuan G. Lu H. Z. Xu X. (2013). Use of natural products as chemical library for drug discovery and network pharmacology. Plos One 8, e62839. 10.1371/journal.pone.0062839 23638153 PMC3636197

[B20] Hilvo M. Denkert C. Lehtinen L. Muller B. Brockmoller S. Seppanen-Laakso T. (2011). Novel theranostic opportunities offered by characterization of altered membrane lipid metabolism in breast cancer progression. Cancer Res. 71, 3236–3245. 10.1158/0008-5472.CAN-10-3894 21415164

[B21] Hoxhaj G. Manning B. D. (2020). The PI3K-AKT network at the interface of oncogenic signalling and cancer metabolism. Nat. Rev. Cancer 20, 74–88. 10.1038/s41568-019-0216-7 31686003 PMC7314312

[B23] Hopperton K. E. Duncan R. E. Bazinet R. P. Archer M. C. (2014). Fatty acid synthase plays a role in cancer metabolism beyond providing fatty acids for phospholipid synthesis or sustaining elevations in glycolytic activity. Exp. Cell Res. 320, 302–310. 10.1016/j.yexcr.2013.10.016 24200503

[B24] Keiser M. J. Roth B. L. Armbruster B. N. Ernsberger P. Shoichet B. K. (2007). Relating protein pharmacology by ligand chemistry. Nat. Biotechnol. 25, 197–206. 10.1038/nbt1284 17287757

[B25] Kim S. Chen J. Cheng T. Gindulyte A. He J. He S. (2019). PubChem 2019 update: Improved access to chemical data. Nucleic Acids Res. 47, D1102–D1109. 10.1093/nar/gky1033 30371825 PMC6324075

[B26] Li F. S. Weng J. K. (2017). Demystifying traditional herbal medicine with modern approach. Nat. Plants 3, 17109. 10.1038/nplants.2017.109 28758992

[B27] Li S. Zhang B. (2013). Traditional Chinese medicine network pharmacology: Theory, methodology and application. Chin. J. Nat. Med. 11, 110–120. 10.1016/s1875-5364(13)60037-0 23787177

[B28] Li X. Chen Z. Li Y. Liang H. Wang H. Li M. (2021). Optical tweezers study of membrane fluidity in small cell lung cancer cells. Opt. Express 29, 11976–11986. 10.1364/oe.420288 33984967

[B29] Li X. H. He X. R. Zhou Y. Y. Zhao H. Y. Zheng W. X. Jiang S. T. (2017). Taraxacum mongolicum extract induced endoplasmic reticulum stress associated-apoptosis in triple-negative breast cancer cells. J. Ethnopharmacol. 206, 55–64. 10.1016/j.jep.2017.04.025 28461119

[B30] Li X. Nakayama K. Goto T. Kimura H. Akamatsu S. Hayashi Y. (2021). High level of phosphatidylcholines/lysophosphatidylcholine ratio in urine is associated with prostate cancer. Cancer Sci. 112, 4292–4302. 10.1111/cas.15093 34328656 PMC8486217

[B50] Liu Y.-J. Shieh P.-C. Lee J.-C. Chen F.-A. Lee C.-H. Kuo S.-C. (2014). Hypolipidemic activity of Taraxacum mongolicum associated with the activation of AMP-activated protein kinase in human HepG2 cells. Food & Funct. 5, 1744–1762. 10.1039/c4fo00183d 24903219

[B31] Mao X. Lei H. Yi T. Su P. Tang S. Tong Y. (2022). Lipid reprogramming induced by the TFEB-ERRα axis enhanced membrane fluidity to promote EC progression. J. Exp. Clin. Cancer Res. 41, 28. 10.1186/s13046-021-02211-2 35045880 PMC8767755

[B22] Martinez M. Poirrier P. Chamy R. Prufer D. SChulze-Gronover C. Jorquera L. (2015). *Taraxacum officinale* and related species-An ethnopharmacological review and its potential as a commercial medicinal plant. J. Ethnopharmacol. 169, 244–262. 10.1016/j.jep.2015.03.067 25858507

[B32] González-Castejón M. Garcia-Carrasco B. Fernandez-Dacosta R. Davalos A. Rodriguez-Casado A. (2013). Reduction of adipogenesis and lipid accumulation byTaraxacum officinale(dandelion) extracts in 3T3L1 adipocytes: Anin vitroStudy. Phytother. Res. 28, 745–752. 10.1002/ptr.5059 23956107

[B33] Miyake T. Parsons S. J. (2012). Functional interactions between Choline kinase α, epidermal growth factor receptor and c-Src in breast cancer cell proliferation. Oncogene 31, 1431–1441. 10.1038/onc.2011.332 21822308 PMC3213328

[B34] Peck B. Schug Z. T. Zhang Q. Dankworth B. Jones D. T. Smethurst E. (2016). Inhibition of fatty acid desaturation is detrimental to cancer cell survival in metabolically compromised environments. Cancer Metab. 4, 6. 10.1186/s40170-016-0146-8 27042297 PMC4818530

[B35] Peck B. Schulze A. (2016). Lipid desaturation - the next step in targeting lipogenesis in cancer? FEBS J. 283, 2767–2778. 10.1111/febs.13681 26881388

[B36] Piñero J. Bravo A. Queralt-Rosinach N. Gutierrez-Sacristan A. Deu-Pons J. Centeno E. (2017). DisGeNET: A comprehensive platform integrating information on human disease-associated genes and variants. Nucleic Acids Res. 45, D833–D839. 10.1093/nar/gkw943 27924018 PMC5210640

[B37] Qu J. Ke F. Liu Z. Yang X. Li X. Xu H. (2022). Uncovering the mechanisms of dandelion against triple-negative breast cancer using a combined network pharmacology, molecular pharmacology and metabolomics approach. Phytomedicine. 99, 153986. 10.1016/j.phymed.2022.153986 35183931

[B38] Saati G. E. Archer M. C. (2011). Inhibition of fatty acid synthase and Sp1 expression by 3, 3′-diindolylmethane in human breast cancer cells. Nutr. Cancer 63, 790–794. 10.1080/01635581.2011.570896 21767081

[B39] Sanchez-Lopez E. Zimmerman T. Gomez del Pulgar T. Moyer M. P. Lacal Sanjuan J. C. CebriAn A. (2013). Choline kinase inhibition induces exacerbated endoplasmic reticulum stress and triggers apoptosis via CHOP in cancer cells. Cell Death Dis. 4, e933. 10.1038/cddis.2013.453 24287694 PMC3847329

[B40] Stelzer G. Rosen N. Plaschkes I. Zimmerman S. Twik M. Fishilevich S. (2016). The GeneCards suite: From gene data mining to disease genome sequence analyses. Curr. Protoc. Bioinforma. 54, 1.30.1–1.30.33. 10.1002/cpbi.5 27322403

[B41] Sun X. Wang M. Wang M. Yu X. Guo J. Sun T. (2020). Metabolic reprogramming in triple-negative breast cancer. Front. Oncol. 10, 428. 10.3389/fonc.2020.00428 32296646 PMC7136496

[B42] Tonje H. Leslie E. Guro G. Tone B. (2017). Metabolic portraits of breast cancer by HR MAS MR spectroscopy of intact tissue samples. Metabolites 7, 18. 10.3390/metabo7020018 PMC5487989 28509845

[B43] Triki M. Rinaldi G. Planque M. Broekaert D. Fendt S. M. Maier C. R. (2020). mTOR signaling and SREBP activity increase FADS2 expression and can activate sapienate biosynthesis. Cell Rep. 31, 107806. 10.1016/j.celrep.2020.107806 32579932 PMC7326293

[B44] Venkatesh H. S. Chaumeil M. M. Ward C. S. Haas-Kogan D. A. James C. D. Ronen S. M. (2012). Reduced phosphocholine and hyperpolarized lactate provide magnetic resonance biomarkers of PI3K/Akt/mTOR inhibition in glioblastoma. Neuro. Oncol. 14, 315–325. 10.1093/neuonc/nor209 22156546 PMC3280799

[B45] Vriens K. Christen S. Parik S. Broekaert D. Yoshinaga K. Talebi A. (2019). Evidence for an alternative fatty acid desaturation pathway increasing cancer plasticity. Nature 566, 403–406. 10.1038/s41586-019-0904-1 30728499 PMC6390935

[B46] Wang C. Kar S. Lai X. Cai W. Arfuso F. Sethi G. (2018). Triple negative breast cancer in Asia: An insider's view. Cancer Treat. Rev. 62, 29–38. 10.1016/j.ctrv.2017.10.014 29154023

[B47] Wang S. Fu J. L. Hao H. F. Jiao Y. N. Li P. P. Han S. Y. (2021). Metabolic reprogramming by traditional Chinese medicine and its role in effective cancer therapy. Pharmacol. Res. 170, 105728. 10.1016/j.phrs.2021.105728 34119622

[B48] Wang X. Shen Y. Wang S. Li S. Zhang W. Liu X. (2017). PharmMapper 2017 update: A web server for potential drug target identification with a comprehensive target pharmacophore database. Nucleic Acids Res. 45, W356–W360. 10.1093/nar/gkx374 28472422 PMC5793840

[B49] Wang X. Wang Z. Y. Zheng J. H. Li S. (2021). TCM network pharmacology: A new trend towards combining computational, experimental and clinical approaches. Chin. J. Nat. Med. 1919, 1–11. 10.1016/s1875-5364(21)60001-8 33516447

[B51] Yamashita Y. Nishiumi S. Kono S. Takao S. Azuma T. Yoshida M. (2017). Differences in elongation of very long chain fatty acids and fatty acid metabolism between triple-negative and hormone receptor-positive breast cancer. Bmc Cancer 17, 589. 10.1186/s12885-017-3554-4 28851309 PMC5576271

[B52] Yin L. Duan J. J. Bian X. W. Yu S. C. (2020). Triple-negative breast cancer molecular subtyping and treatment progress. Breast Cancer Res. 22, 61. 10.1186/s13058-020-01296-5 32517735 PMC7285581

[B53] Yoshida G. J., (2015). Metabolic reprogramming: The emerging concept and associated therapeutic strategies. J. Exp. Clin. Cancer Res. 34, 111–210. 10.1186/s13046-015-0221-y 26445347 PMC4595070

[B54] Zhang B. Wu J. Guo P. Wang Y. Fang Z. Tian J. (2020). Down-regulation of SREBP via PI3K/AKT/mTOR pathway inhibits the proliferation and invasion of non-small-cell lung cancer cells. Onco. Targets. Ther. 13, 8951–8961. 10.2147/ott.S266073 32982287 PMC7490059

[B55] Zhang H. W. Lv C. Zhang L. J. Guo X. Shen Y. W. Nagle D. G. (2021). Application of omics- and multi-omics-based techniques for natural product target discovery. Biomed. Pharmacother. 141, 111833. 10.1016/j.biopha.2021.111833 34175822

[B56] Zhang R. Zhu X. Bai H. Ning K. (2019). Network pharmacology databases for traditional Chinese medicine: Review and assessment. Front. Pharmacol. 10, 123. 10.3389/fphar.2019.00123 30846939 PMC6393382

[B57] Zhong Z. Qiang W. W. Tan W. Zhang H. Wang S. Wang C. (2016). Chinese herbs interfering with cancer reprogramming metabolism. Evid. Based. Complement. Altern. Med. 2016, 9282813–9282910. 10.1155/2016/9282813 PMC4875995 27242914

[B58] Zhu H. Zhao H. Zhang L. Xu J. Zhu C. Zhao H. (2017). Dandelion root extract suppressed gastric cancer cells proliferation and migration through targeting lncRNA-CCAT1. Biomed. Pharmacother. 93, 1010–1017. 10.1016/j.biopha.2017.07.007 28724210

[B59] Zou Y. Huang L. Sun S. Yue F. Li Z. Ma Y. (2021). Choline kinase alpha promoted glioma development by activating PI3K/AKT signaling pathway. Cancer Biother. Radiopharm. 10.1089/cbr.2021.0294 34788108

