# Supplementary material for: Dandelion extract inhibits triple-negative breast cancer cell proliferation by interfering with glycerophospholipids and unsaturated fatty acids metabolism
Source: Front Pharmacol. 2022 Sep 6;13:942996. doi: 10.3389/fphar.2022.942996 (PMC9486077; doi:10.3389/fphar.2022.942996)
Supplement: Supplementary file 1 [file DataSheet1.docx]

**Supplementary Material**

**Dandelion extract inhibits triple-negative breast cancer cell proliferation by interfering with glycerophospholipids and unsaturated fatty acids metabolism**

Shan Wang^1, 2^, Huifeng Hao^2^, Yan-Na Jiao^2^, Jia-Lei Fu^2^, Zheng-Wang Guo^2^, Yang Guo^2^, Yuan Yuan^2^, Ping-Ping Li*, Shu-Yan Han^1, 2,^ *

1. Department of Integration of Chinese and Western Medicine, School of Basic Medical Sciences, Peking University, Beijing, China

2. Key Laboratory of Carcinogenesis and Translational Research (Ministry of Education), Department of Integration of Chinese and Western Medicine, Peking University Cancer Hospital and Institute, Beijing, China

**1. Supplementary Data**

**1.1. Quantitative proteomics analysis**

1) *Total protein extract and trypsin digestion.* All cell samples were resuspended in the lysis buffer (8M urea, 1% protease inhibitor cocktail) and sonicated at 4°C three times. Subsequently, centrifugation was conducted to remove the remaining debris at 12000 g and 4 °C for 10 min, and the supernatant was collected to measure the protein concentration by BCA kit (Scientz, China). Equal amounts of protein samples were taken, and the appropriate amount of standard protein was added for trypsin digestion. Then lysis buffer was added to ensure the volume of each sample was consistent. Afterward, the protein solution was reduced with 5 mM dithiothreitol for 30 min at 56 °C and alkylated with 11 mM iodoacetamide for 15min at room temperature in darkness. Then, the urea concentration of protein solution was diluted to less than 2 M by adding 100 mM triethylammonium bicarbonate (TEAB). Then, trypsin was added at a ratio of 1:50 (trypsin: protein) for the first digestion overnight and 1:100 (trypsin: protein) for a second 4 h digestion.

2) *TMT labeling and HPLC fractionation.* The peptides were desalted by Strata X C_18_ SPE column (Phenomenex, USA) and dried in a vacuum. Then the peptides were reconstituted in 0.5 M TEAB and processed with the TMT labeling kit (Scientz, China). The cell samples treated with dandelion extract were labeled with 126, 127, 128 tags, and samples of vehicle treatment were labeled with 129, 130, 131 tags. The peptide mixtures were incubated for 2 hours at room temperature and pooled, desalted, and dried by vacuum centrifugation. The tryptic peptides were fractionated into 60 fractions with an 8%-32% acetonitrile gradient (pH 9.0) for 60min by high pH reverse-phase HPLC with a C_18_ column (Thermo Betasil, 5 μm particles, 10 mm inner diameter, and 250 mm length). Then, the eluted peptides were combined into 6 fractions and dried by vacuum centrifuging.

3) *LC-MS/MS analysis*. The peptides were dissolved in 0.1% formic acid (solvent A), and the mixtures were directly loaded onto a reversed-phase analytical column (15 cm length, 75 μm inner diameter). The chromatographic condition was set as an increase of 6%-23% solvent B (0.1% formic acid in 98% acetonitrile) over 26 min, 23%-15% in 8min, climbing to 80% in 3min, and then holding at 80% for the last 3 min with a constant flow rate of 400 nL/min in the EASY-nLC 1000 UPLC system (Thermo Scientific, USA). Afterward, the peptides were subjected to a nanospray ion (NSI) source followed by tandem mass spectrometry in Q Exactive Plus (Thermo Scientific, USA) coupled online to the UPLC. The electrospray voltage was 2.0 kV. The ion data of MSI ranging from 400 to 1500 Da was detected in the Orbitrap at a resolution of 120000. The ion data of MS2 over 100Da was detected in the Orbitrap at a resolution of 15000. The data collection was performed via a data-dependent procedure that alternated between one MS scan followed by 20 MS/MS scans with 15.0s dynamic exclusion.

4) *Database search.* The raw MS/MS data were processed by Maxquant software (version 1.5.2.8) and searched in the human UniProt database. Trypsin/P was specified as a cleavage enzyme allowing up to 4 missing cleavages. The mass tolerance for precursor ions was set as 20 ppm in the First search and 5 ppm in the Main search, and the mass tolerance for fragment ions was set as 0.02 Da. The fixed modification was carbamidomethyl, and variable modifications were oxidation and acetylation. False discovery rate (FDR) was adjusted to < 1%, and the minimum score for modified peptides was set > 40.

**1.2. Untargeted metabolomics analysis**

Untargeted metabolomics was analyzed using an ultra-performance liquid chromatography-electrospray ionization-tandem mass spectrometry system (UPLC-ESI-MS/MS; UPLC, ExionLC AD; MS, QTRAP@). The steps are as follows.

1) *Cell sample preparation.* All MDA-MB-231 cell samples were added 1 mL pre-cooled 80% methanol aqueous solution and whirled for 2 min. Then, freeze the mixture for 3 minutes in liquid nitrogen and thaw for 5 minutes three times. Finally, centrifuge the mixture for 10 min (4°C, 12000r/min) and transfer 200 μL of supernatant into the inner liner of the corresponding injection bottle for further analysis.

2) *UPLC conditions.* Column: Waters ACQUITY UPLC HSS T3 C_18_ (1.8μm, 2.1mm*100mm). column temperature, 40°C; flow rate, 0.4 mL/min; injection volume, 2μL; solvent system, water (0.1% formic acid): acetonitrile (0.1% formic acid). gradient program: 95:5 v/v at 0 min, 10:90 v/v at 10.0 min, 10:90 v/v at 11.0 min, 95:5 v/v at 11.1 min, and 95:5 v/v at 14.0 min.

3) *ESI-MS/MS analysis*. Triple quadrupole-linear ion trap mass spectrometer LC-MS/MS system equipped with an ESI Turbo Ion-Spray was performed in positive and negative ion mode. The ESI parameters were as follows: source temperature, 500 °C; ion spray voltage, 5500 V (positive), -4500 V (negative); ion source gas I, 50 psi; gas II, 60 psi; curtain gas, 25 psi; collision gas, high. The qualitative analysis of the metabolites was performed by the retention time and sub-ion pairs based on the metware database, and the quantification was calculated by multiple reaction detection mode analysis and its integral correction.

4) *Data processing and analysis.* The raw data was collected and analyzed by Analyst software (Sciex, version 1.6.3). The stability and repeatability of samples were evaluated by the unsupervised principal component analysis (PCA) and supervised orthogonal partial least squared discriminant analysis (OPLS-DA). Significantly regulated metabolites between groups were determined by variable importance (VIP) ≥ 1 and absolute Log2FC (fold change) ≥ 1. VIP values were extracted from the OPLS-DA result, which contains score plots, and permutation plots were generated using the R package MetaboAnalystR.

**1.3. Untargeted lipidomics**

Untargeted lipidomics was performed using UPLC-MS/MS. All MDA-MB-231 cell samples were added 1 mL pre-cooled methanol and sonicated for 0.5h. Subsequently, centrifugation was performed for 10 min (4°C, 12000r/min), and 95 μL of supernatant was added to 5μL of internal standard solution (Cer/Sph) and mixed well. The mixture was transferred to the injection bottle for further analysis.

Waters ACQUITY UPLC I-Class system was used for chromatographic separation. The condition of targeted lipid chromatography was as follow: column, Waters UPLC BEH C_8_ (1.7 μm, 2.1mm*100 mm); column temperature, 55°C; flow rate, 0.26 mL/min; injection volume, 5μL; mobile phases, solvent A (acetonitrile/water, 6:4, v/v) and solvent B (isopropanol/acetonitrile, 9:1, v/v), both containing 0.1% formic acid and 10 mM ammonium formate. gradient program: 68:32 v/v at 0 min, 68:32 v/v at 1.5 min, 15:85 v/v at 15.5 min, 3:97 v/v at 15.6 min, 3:97 v/v at 18.0 min, 68:32 v/v at 18.1 min, and 68:32 v/v at 20.0 min. Moreover, the condition of targeted fatty acid chromatography was as follow: column, Waters UPLC BEH C_8_ (1.7 μm, 2.1mm*100 mm); column temperature, 55°C; flow rate, 0.26 mL/min; injection volume, 5μL; mobile phases, solvent A (acetonitrile/water, 1:10, v/v, 0.1% acetic acid, 1 mM ammonium acetate) and solvent B (isopropanol/acetonitrile, 1:1, v/v). gradient program: 90:10 v/v at 0 min, 65:35 v/v at 2 min, 65:35 v/v at 2 min, 15:85 v/v at 4 min, 0:100 v/v at 6 min, 0:100 v/v at 7.5 min, 90:10 v/v at 7.6 min, and 90:10 v/v at 9 min.

A Waters XEVO TQ-S Micro tandem quadrupole mass spectrometry system was used for mass spectrometry analysis. The setting parameters were as follows: ESI^+^, spray voltages: 3.7kV, temperature, 150°C; ESI^-^, spray voltages: 3.5kV, temperature, 150°C; desolventizing temperature, 350°C; desolventizing gas flow rate, 1000L/h; cone hole voltage, 29.0V; gas flow rate. 150 L/h; sheath gas (N2): 30 psi, auxiliary gas (N2): 10 psi, heated capillary temperature: 320 °C, resolution: 70000, m/z range: 50-1500Da. The peak area of raw data was calculated using TargetLynx quantitative software with an allowable error of 15s. The analysis was consistent with the metabolomics.

**2. Supplementary figures**

**
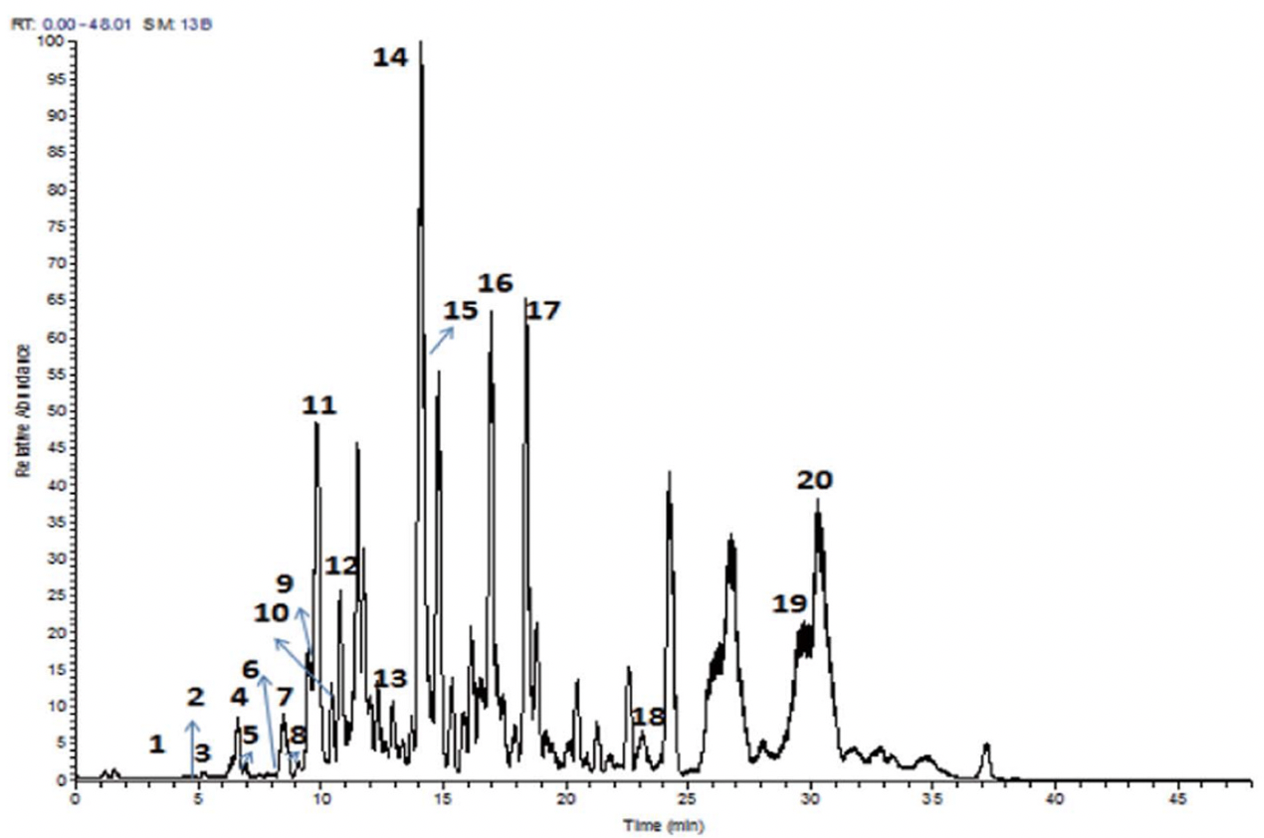
**

**Fig. S1.** Identification of Compounds form the dandelion extract via UHPLC-ESI-Orbitrap MS/MS chromatogram in negative mode. (1) vanillic acid; (2) caftaric acid; (3) hydroxybenzoic acid; (4) esculetin; (5) caffeic acid; (6) hesperidin; (7) syringic acid; (8) p-coumaric acid; (9) luteolin-7-O-β-D-rutinoside; (10) isorhamnetin 3-β-D-glucoside; (11) luteolin-7-O-β-D-glucoside; (12) chlorogenic acid; (13) ferulic acid; (14) luteolin; (15) quercetin; (16) 10,15-octadecadienoic acid; (17) 9, 10,11-trihydroxy-(12Z)-octadecanienoic acid; (18) 9, 10,11-trihydroxy-9,11-octadecadienoic acid; (19) 9-hydroxy-10,12,15-Octadecatrienoic acid; (20) picrasinoside F.

**
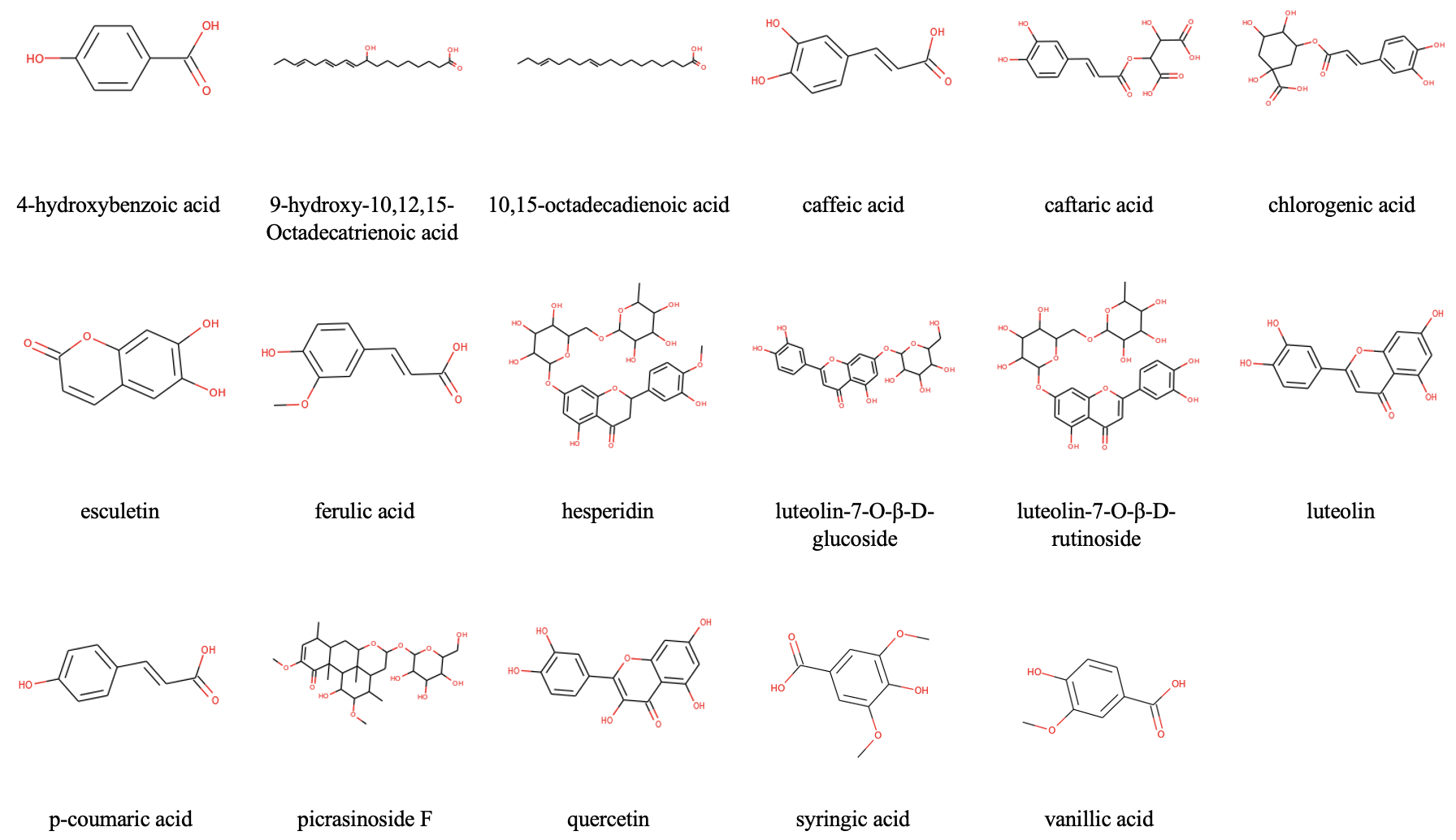
**

**Fig. S2.** The chemical structures of 17 compounds in network pharmacological analysis.


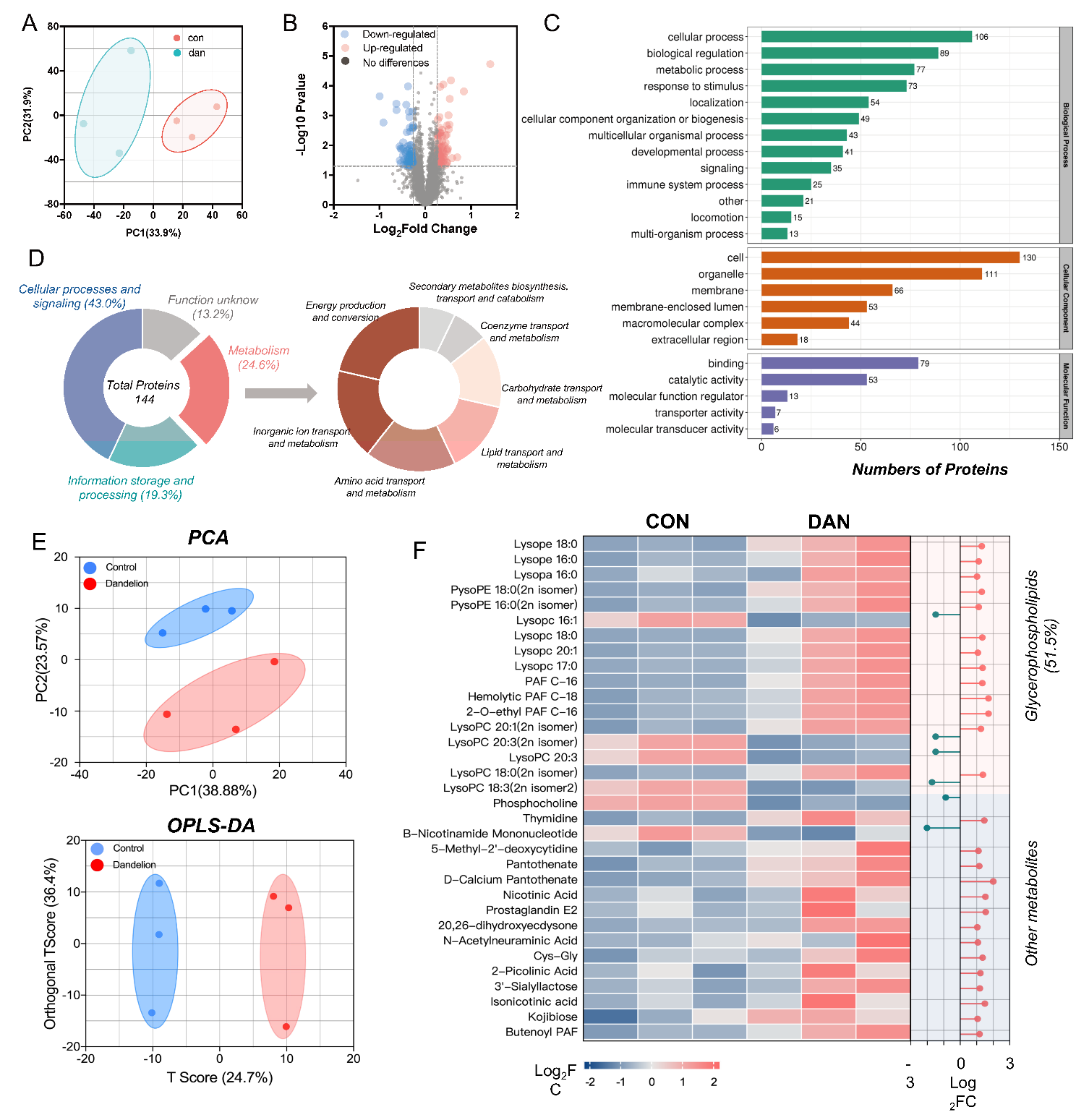


**Fig. S3. Proteome profiles of MDA-MB-231 cells after dandelion extract treatment.** (**A**). The PCA of cell samples. (**B**) The volcano plot of differentially expressed proteins. (**C**) GO function enrichment of differentially expressed proteins. (**D**) The functional clusters of orthologous groups of the differentially expressed proteins. (**E**) The PCA and OPLS-DA analysis of the six cell samples in untargeted metabolomics. (**F**) The heatmap and change dumbbell charts of the significantly changed metabolites of untargeted metabolomics.


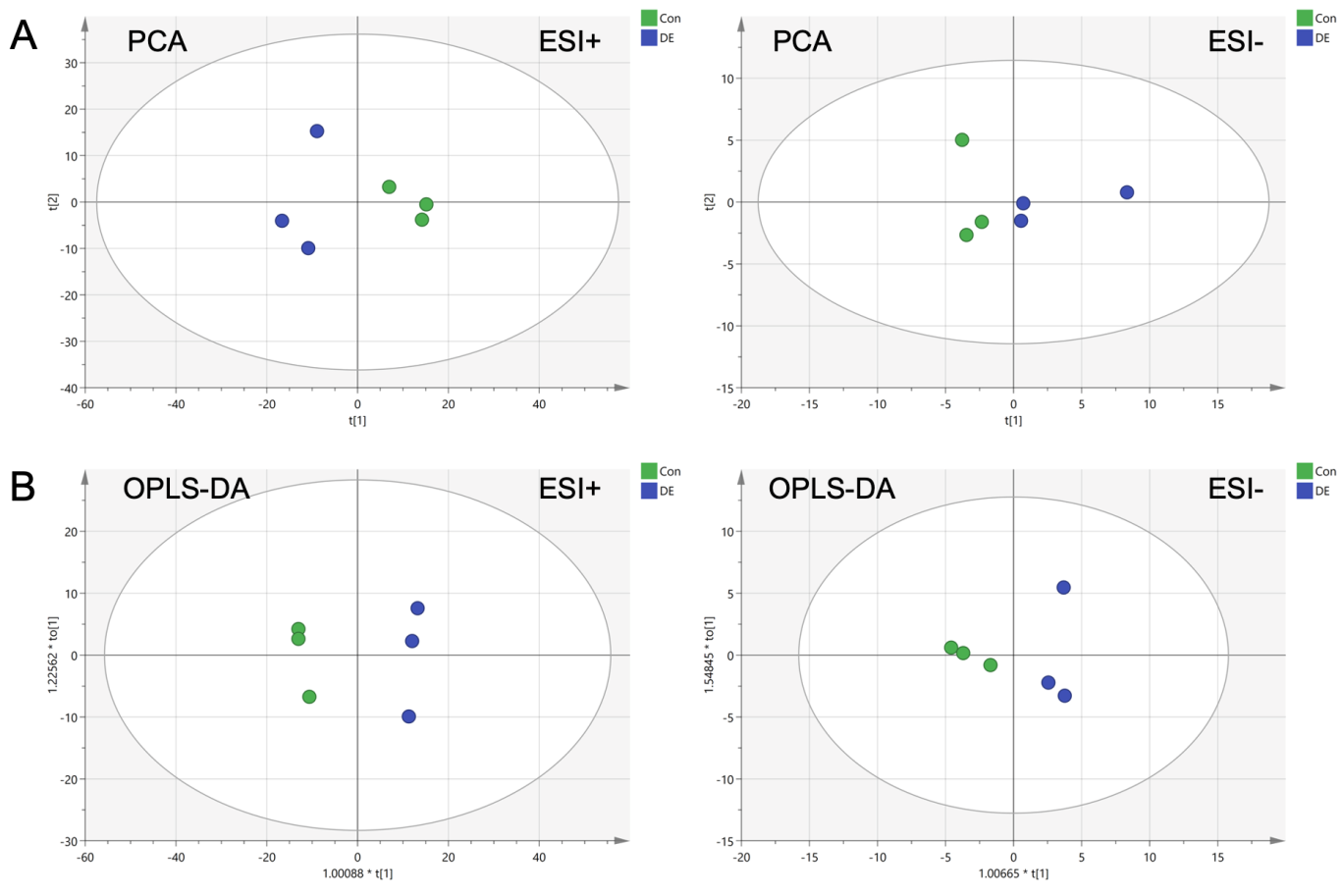


**Fig. S4.** The multivariate statistical analysis of the MDA-MB-231 cell samples of lipidomics. PCA: Principal Component Analysis; OPLS-DA: orthogonal partial least-squares discrimination analysis.


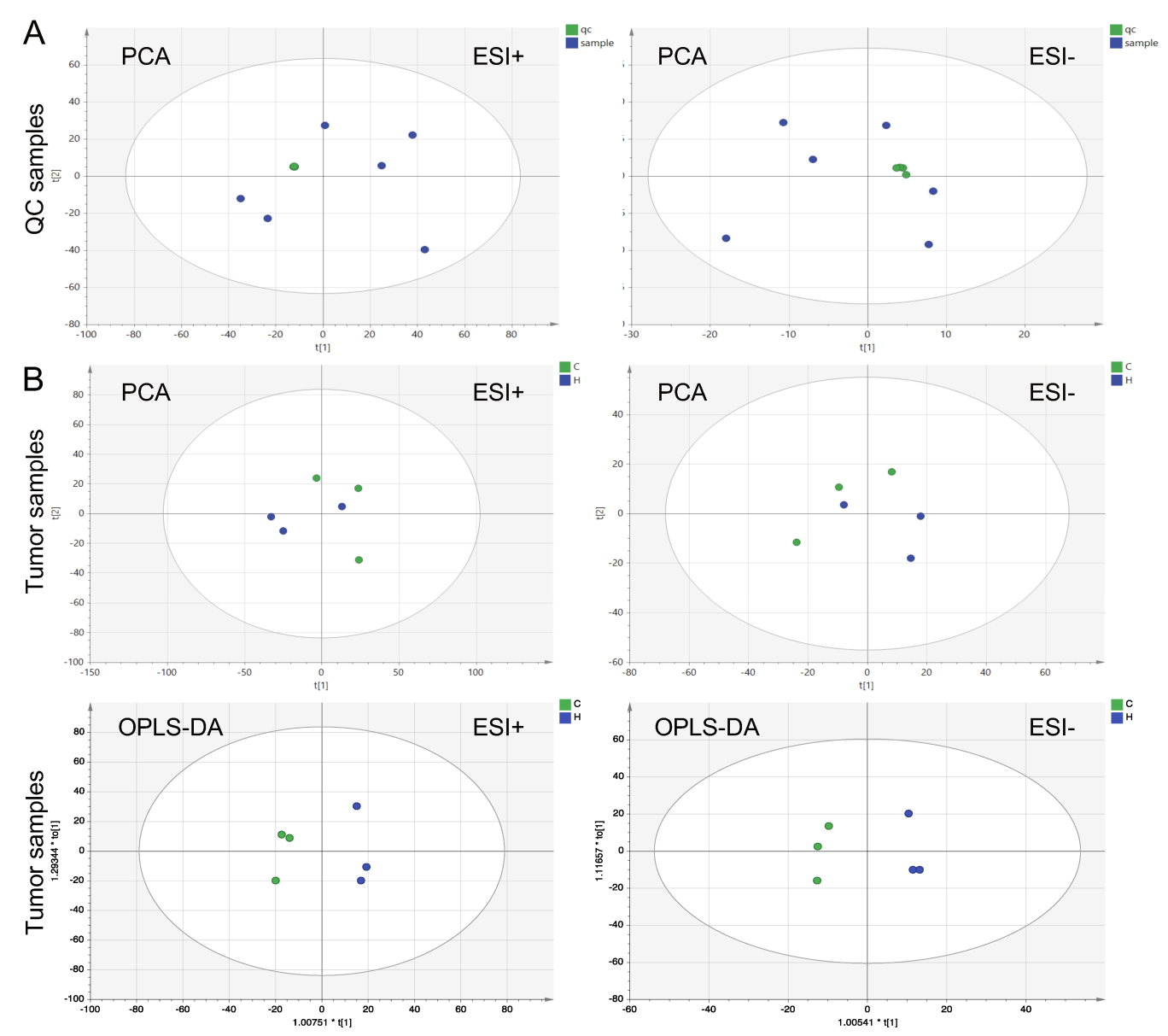


**Fig. S5.** The multivariate statistical analysis of the TNBC tumor tissue samples of lipidomics. PCA: Principal Component Analysis; OPLS-DA: orthogonal partial least-squares discrimination analysis.


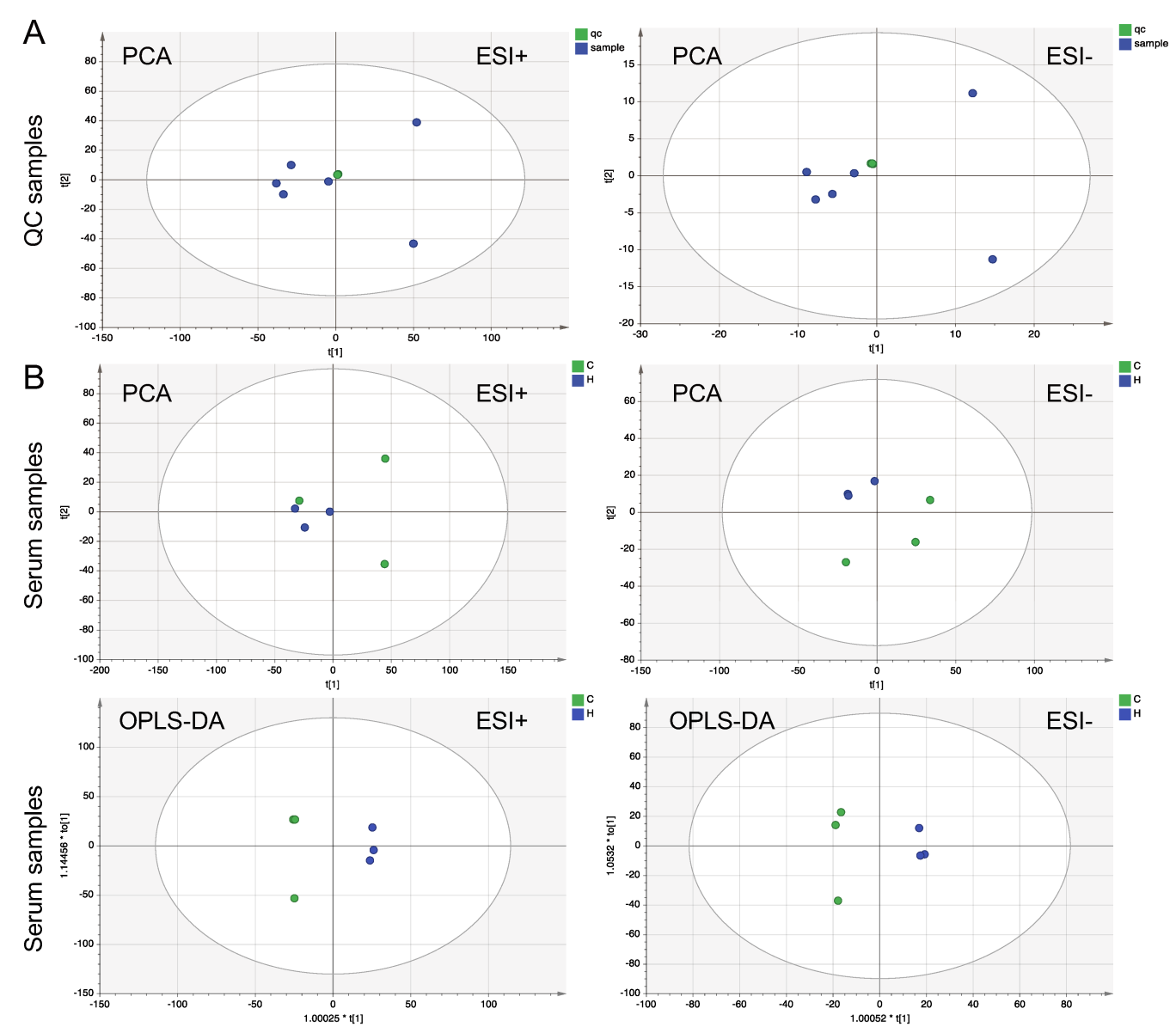


**Fig. S6.** The multivariate statistical analysis of the plasma samples of lipidomics. PCA: Principal Component Analysis; OPLS-DA: orthogonal partial least-squares discrimination analysis.


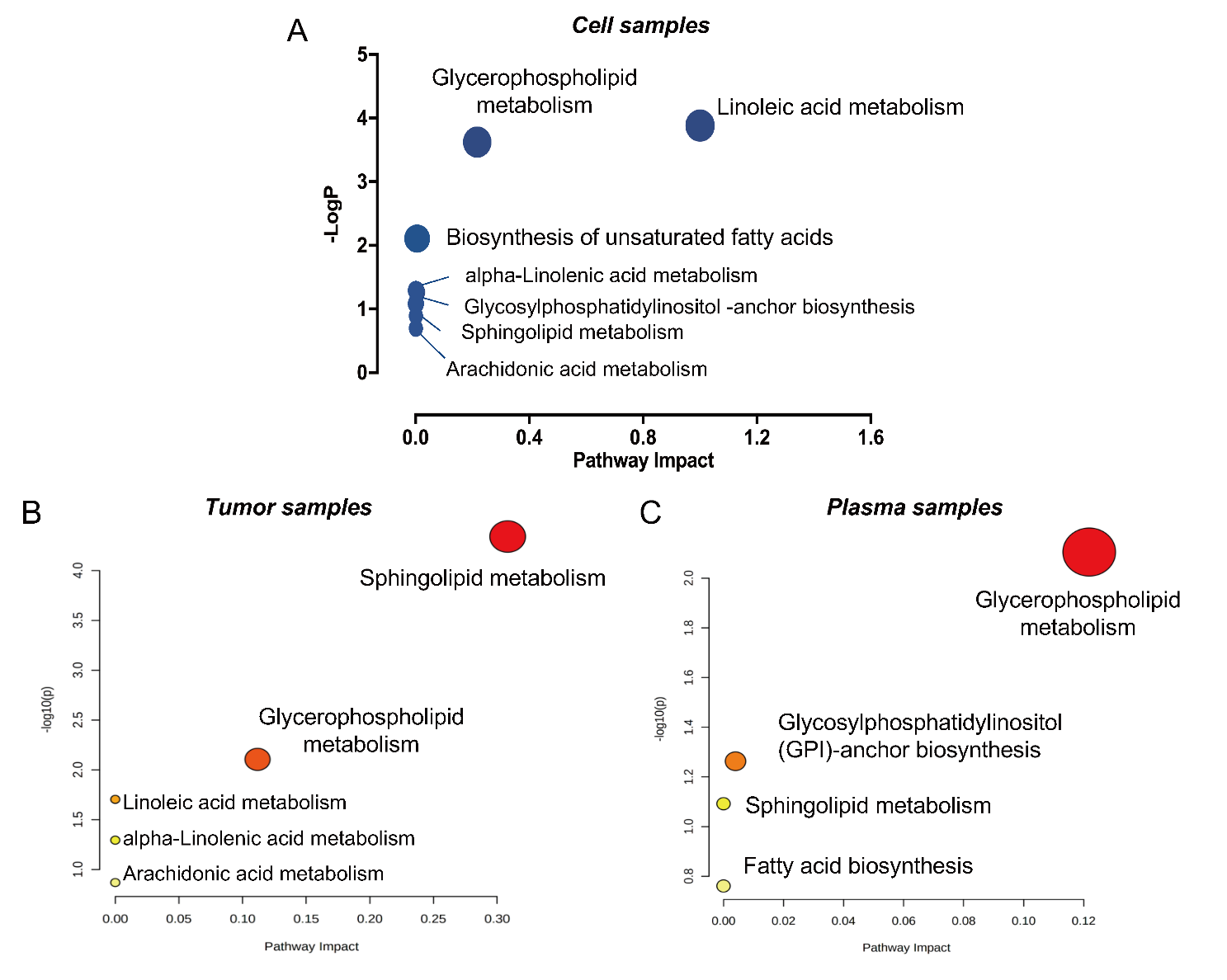


**Fig. S7.** The KEGG analysis of the significantly changed metabolites by lipidomics.

**2. Supplementary tables**

**Table S1.** The primers sequences used in this study.

**Table S2.** The specific information of the antibodies used in this study.

**Table S3.** The detail information 17 bioactive compounds from the dandelion extract.

**Table S4.** The overlapping targets between the compounds form danelion extract and TNBC.

**Table S5.** Topological parameters of the compounds in "compound-disease-target" network.

**Table S6.** The GO functional enrichement of the overlapping targets.

**Table S7.** The KEGG pathway enrichment of the overlapping targets.
